# A Symmetric Cogeneration Fuel Cell for Coupled Production of Hydrogen, Ammonia and Formate

**DOI:** 10.1002/advs.75826

**Published:** 2026-05-25

**Authors:** Yingjie Song, Peimiao Zou, Renhang Wang, Qi Zhang, Yisong Han, Christopher Waldron, Marc Walker, Ben G. Breeze, Shanwen Tao

**Affiliations:** ^1^ School of Engineering University of Warwick Coventry UK; ^2^ Hartnoll Centre University of Warwick Coventry UK; ^3^ Department of Physics University of Warwick Coventry UK; ^4^ Research Technology Platforms University of Warwick Coventry UK

**Keywords:** cogeneration fuel cell, electrochemical synthesis, formaldehyde oxidation reaction, nitrate reduction reaction, symmetric fuel cell

## Abstract

Electrochemical synthesis offers a sustainable route to convert abundant feedstocks into value‐added chemicals under mild conditions and couple chemical manufacturing with renewable electricity. However, its practical impact is often limited by system‐level energy inefficiency, motivating rational reaction pairing to reduce energy input while upgrading product value. Here, we develop a symmetric cogeneration fuel cell that couples one‐electron formaldehyde oxidation reaction with nitrate reduction to co‐produce hydrogen, ammonia and formate while generating electricity. This symmetric cell was enabled by a FOR and NO_3_RR bifunctional Cu nano leaves coated with Co(OH)_2_ catalyst, which constructs an interfacial hydrogen network capable of synchronizing NO_3_^–^ reduction with active hydrogen delivery. As a result, the catalyst achieves an industrial current density of 2.35 A cm^−2^ at −1 V vs RHE with a high Faradaic efficiency of 96.38%. Operating at 35°C, the symmetric cell delivers a peak power density of 13.24 mW cm^−2^, representing the highest value reported to date for unassisted ammonia synthesis systems. This work establishes a simple yet versatile strategy for integrating value‐added chemical synthesis with electricity generation and offers a generalizable framework for other paired electrosynthesis processes.

## Graphical abstract

A symmetric cogeneration fuel cell integrating value‐added chemical synthesis with electricity generation by coupling formaldehyde oxidation with the nitrate reduction was developed. This work highlights the broader potential of symmetric cogeneration fuel cells as a general platform for rational reaction pairing, offering a practical pathway to lower energy input, upgrade product value and advance electrochemical synthesis driven by renewable electricity.

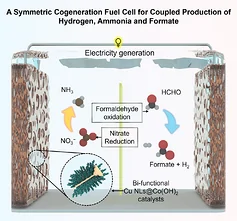

## 1 Introduction

Electrochemical synthesis provides a new approach to connecting renewable electricity with sustainable chemical manufacturing, enabling the conversion of abundant feedstock into value‐added products under mild conditions [1, 2]. However, many electrochemical synthesis processes remain constrained by system‐level inefficiency, as the emphasis is typically placed on a single target reaction, while the counter electrode is underutilized or assigned to low‐value reactions. To address this imbalance, coupling alternative anodic and cathodic reactions has emerged as an effective strategy to enhance overall energy efficiency and product value [3, 4, 5]. For instance, in water electrolysis and electrochemical conversion of carbon‐ and nitrogen‐containing molecules, the overall performance of two‐electrode electrochemical systems is fundamentally constrained by the oxygen evolution reaction (OER) [6, 7, 8]. The OER at anode significantly suffers from sluggish kinetics, requiring high anodic over‐potential while producing low‐value O_2_, thereby limiting both energy efficiency and economic viability [9, 10, 11]. Replacing OER with the oxidation of small organic molecules has therefore emerged as an attractive strategy to reduce cell voltage while simultaneously generating value‐added products [12, 13].

Beyond improving the energy efficiency of electrochemical synthesis, reaction coupling can be extended toward cogeneration fuel cells, in which chemicals harvesting is integrated with electricity production [14, 15]. In such systems, chemical energy stored in redox‐active molecules is simultaneously converted into value‐added products and electricity, offering a compelling alternative to conventional, electricity‐consuming electrolysis and fuel cells with low value products. Within the framework of cogeneration fuel cells, the key challenge lies in identifying redox couples that combine favorable thermodynamics with value‐added products at both electrodes. Among the many possible candidates, coupling electrochemical nitrate reduction (NO_3_RR) to ammonia (NH_3_) with the one‐electron formaldehyde oxidation reaction (FOR) represents a particularly compelling reaction pair. Under alkaline conditions (pH 14), the thermodynamic equilibrium potential for nitrate reduction to ammonia is +0.69 V vs RHE: [16, 17, 18]

(1) $$\begin{array}{c} N{O_{3}}^{-}+6H_{2}O\;+8e^{-}\to NH_{3}+\;9OH^{-}\to E^{\circ }=+0.69\;V\;\mathrm{vs}\;\mathrm{RHE} \end{array}$$

In contrast, the equilibrium potential of the one‐electron FOR in alkaline media (pH 14) is −0.22 V vs RHE: [19, 20, 21]

(2) $$\begin{array}{ccc} 2\mathrm{HCHO}\;+4OH^{-} & \to & 2\mathrm{HCO}O^{-}+H_{2}+2H_{2}O+\;2e^{-}\\ & \to & E^{\circ }=-\;0.22\;V\;\mathrm{vs}\;\mathrm{RHE} \end{array}$$

Accordingly, coupling these two half‐cell reactions yields a theoretical open‐circuit voltage (OCV) of 0.91 V (+0.69 V – (−0.22 V) = +0.91 V) at pH 14. The positive cell voltage indicates that the NO_3_RR||FOR configuration is thermodynamically spontaneous, enabling electricity generation rather than consumption during simultaneous hydrogen, ammonia, and formate production (Figure S1).

Despite favorable redox matching, cogeneration fuel cells require robust electrocatalysts delivering high activity, selectivity, and durability for both half‐reactions. In this regard, symmetric electrochemical architectures, where the same catalytic material is deployed on both sides, offer distinct advantages in manufacturability and scalability by eliminating the need for two different electrodes and streamlining fabrication and assembly [22, 23, 24, 25]. Therefore, developing bi‐functional catalysts and symmetric cogeneration fuel cells is of considerable significance and holds strong potential as a general strategy to integrate value‐added chemical synthesis with electricity generation within a single electrochemical platform.

Here, we introduce a symmetric cogeneration fuel cell that simultaneously produces hydrogen, ammonia, formate and electricity based on a core‐shell array catalyst composed of copper nano‐leaves coated with cobalt hydroxide nanosheets (Cu NLs@Co(OH)_2_). The Cu nano leaves (NLs) core enables rapid reduction of NO_3_^−^ into NO_2_^−^ intermediates, whereas the Co(OH)_2_ shell accelerates interfacial water dissociation, locally generates H^*^ and facilitates H^*^ spillover to the underlying Cu NLs for further conversion into ammonia. This H^*^ generating‐consuming balance at the Cu‐Co(OH)_2_ interface boosts efficient NO_3_RR, achieving a high current density of 2.35 A cm^−2^ at −1 V vs RHE with a high ammonia selectivity (FE = 96.38%). Beyond NO_3_RR, Cu NLs@Co(OH)_2_ also exhibits excellent activity toward the FOR. When paired cathodic NO_3_RR and anodic FOR, the bifunctional catalyst Cu NLs@Co(OH)_2_ enables a symmetric cogeneration fuel cell, which co‐produces hydrogen, ammonia, formate and electricity. This symmetric cogeneration fuel cell achieved an open‐circuit voltage of 0.80 V, a peak power density of 13.24 mW cm^−2^ at 35°C, with stable performance during the measured 120 h. Compared with conventional NO_3_RR paired with OER, our NO_3_RR||FOR coupling configuration lowers the cost of energy input and upgrades the anodic products, which deliver a potential net profit of $39575.43 per ton ammonia from co‐produced formate, H_2_ and electricity.

## 2 Results and Discussion

### 2.1 Synthesis of Cu NLs@Co(OH)_2_ Catalysts

**FIGURE 1:**
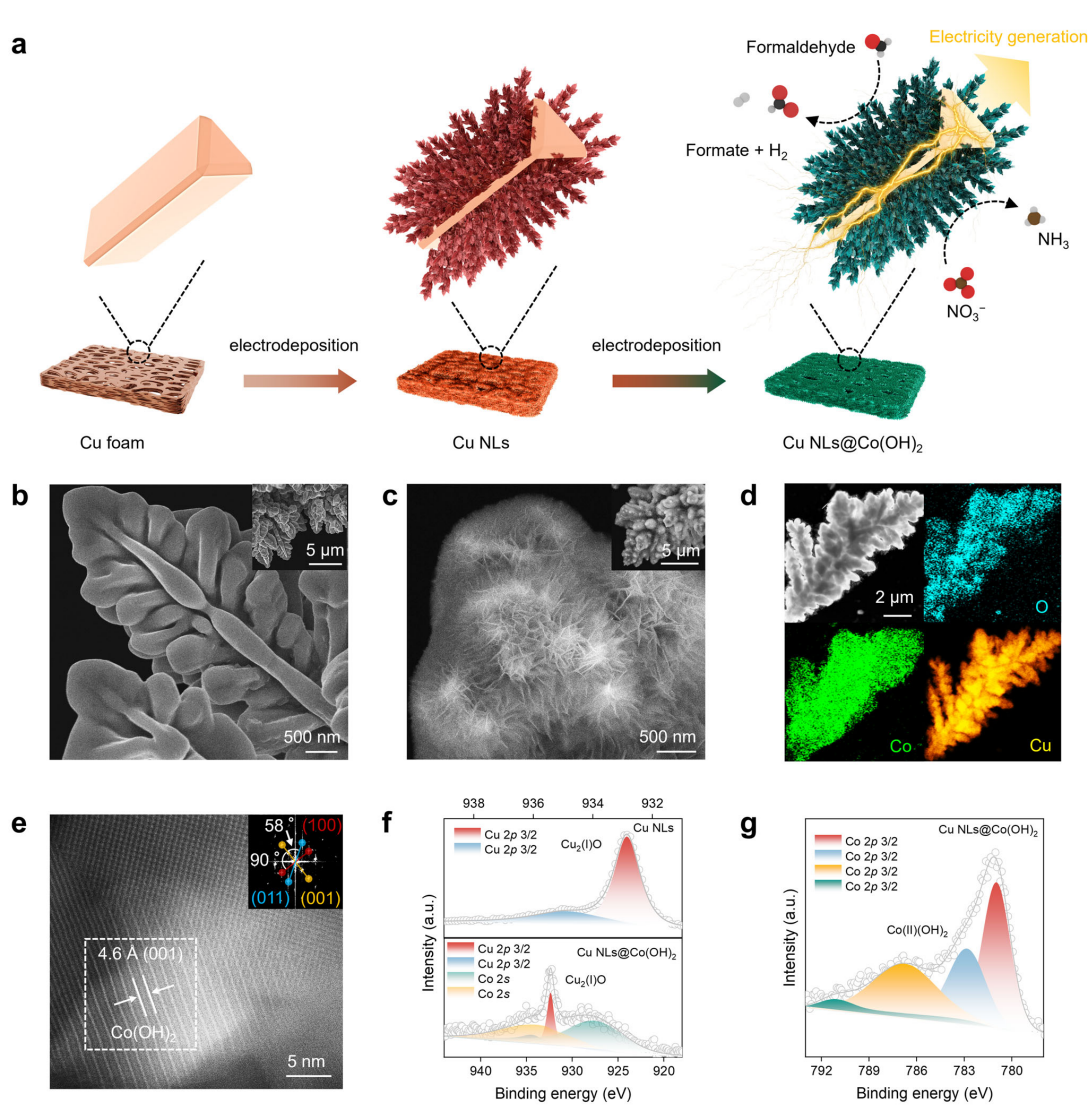
Synthesis of Cu NLs@Co(OH)_2_ catalysts. (a) Schematic illustration of the synthesis of Cu NLs@Co(OH)_2_ catalysts. (b) SEM images of Cu NLs. (c) SEM images of Cu NLs@Co(OH)_2_. (d) ADF‐STEM image and corresponding EDX elemental maps of Cu NLs@Co(OH)_2_. (e) High‐resolution ADF‐STEM image of Cu NLs@Co(OH)_2_ (inset: corresponding FFT of the image). (f) High‐resolution XPS spectra in the Cu 2*p*_3/2_ region of Cu NLs and Cu NLs@Co(OH)_2_ (including the Co 2*s* contribution). (g) High‐resolution XPS spectra in the Co 2*p*_3/2_ region of Cu NLs@Co(OH)_2_.

The synthesis of the bifunctional Cu NLs@Co(OH)_2_ catalyst is schematically illustrated in Figure 1a. Briefly, the vertically aligned copper nano leaves (Cu NLs) array was first grown on the skeleton of bare Cu foam, followed by the conformal electrodeposition of Co(OH)_2_ layer onto the Cu NLs surface. Scanning electron microscopy (SEM) images reveal that the pristine Cu NLs exhibit a vertically oriented nano leaves architecture with wrinkled surfaces (Figure 1b). After the second electrodeposition step, the Cu NLs core is uniformly encapsulated by an ultrathin nanosheet shell (Figure 1c). Annular dark‐field scanning transmission electron microscopy (ADF‐STEM) images (Figure S2), together with the corresponding energy‐dispersive X‐ray spectroscopy (EDX) elemental maps (Figure 1d and Figure S3), confirm the core‐shell configuration and the homogeneous distribution of Cu and Co throughout the Cu NLs@Co(OH)_2_ architecture. High‐resolution ADF‐STEM images and their fast Fourier transform (FFT) patterns further resolve the (001), (100), and (011) lattice planes of Co(OH)_2_, evidencing the successful formation of the Co(OH)_2_ nanosheet shell.

X‐ray diffraction (XRD) patterns of both Cu NLs and Cu NLs@Co(OH)_2_ display prominent diffraction peaks at 43.3° and 50.4°, corresponding to metallic Cu (Figure S4). Additional peaks at 36.6° and 42.5° are assigned to the (111) and (200) planes of Cu_2_O, arising from slight surface oxidation of Cu NLs upon air exposure [26, 27]. X‐ray photoelectron spectroscopy (XPS) was employed to further probe the surface electronic structure. As shown in Figure 1f, two peaks at the binding energy of 932 eV and 935 eV for both Cu NLs and Cu NLs@Co(OH)_2_ can be ascribed to Cu 2*p*_3/2_ photoemission from the Cu_2_O on the surface of Cu NLs [28, 29], which is consistent with the XRD results. Additional contributions in the Cu 2*p*_3/2_ region were observed for the CuNLs@Co(OH)2 due to the overlapping Co 2*s* peaks, providing preliminary evidence for Co species at the surface. The Co 2*p*_3/2_ spectrum recorded from Cu NLs@Co(OH)_2_ (Figure 1g), strongly correlates with reference spectra of Co(OH)_2_ in literature [29, 30]. In addition, the decrease of the Cu relative abundance at the surface from 21 atomic % to 1 atomic % (Table S1), and commensurate reduction in the metal oxide peak in the O 1*s* region (Figure S5) also indicates the successful coating of Co(OH)_2_ on the surface of Cu NLs.

### 2.2 NO_3_RR Performance of Cu NLs@Co(OH)_2_

**FIGURE 2:**
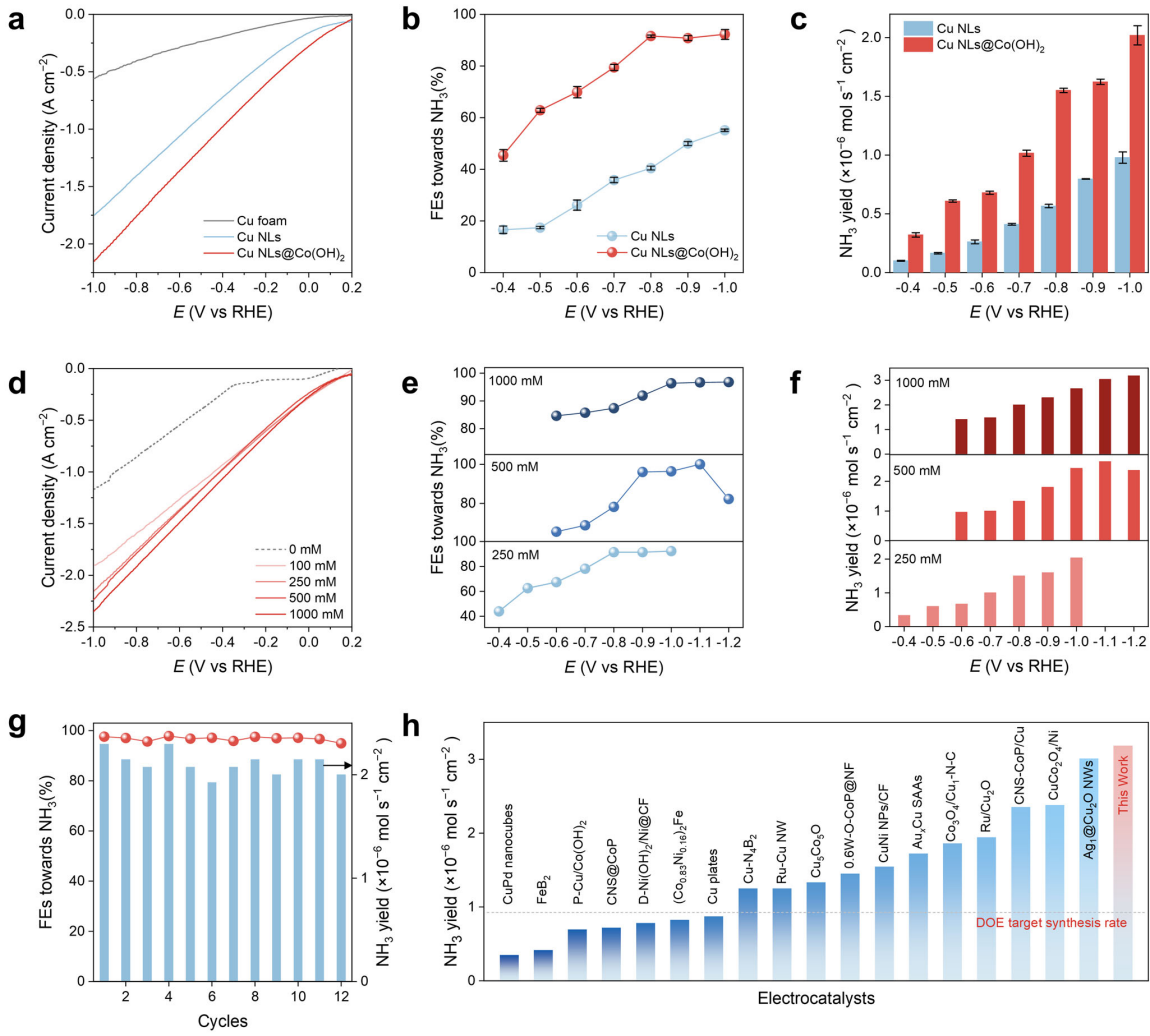
NO_3_RR performance of Cu NLs@Co(OH)_2_. (a) linear sweep voltammetry (LSV) curves of bare Cu foam, Cu NLs and Cu NLs@Co(OH)_2_ in 1 M KOH with 250 mM NO_3_^−^. (b) FEs toward NH_3_ of Cu NLs and Cu NLs@Co(OH)_2_ in 1 M KOH with 250 mM NO_3_^−^. (c) NH_3_ yield rate of Cu NLs and Cu NLs@Co(OH)_2_ in 1 M KOH with 250 mM NO_3_^−^. (d) LSV curves, (e) FEs toward NH_3_ and (f) NH_3_ yield rate of Cu NLs@Co(OH)_2_ in 1 M KOH with varying NO_3_^−^ concentration. (g) FEs toward NH_3_ and yield rates during 12 continuous cycles for Cu NLs@Co(OH)_2_ at −1 V vs RHE in 1 M KOH with 250 mM NO_3_^−^. (h) Comparison of NH_3_ yield rate of Cu NLs@Co(OH)_2_ with those of previous reported electrocatalysts.

The NO_3_RR performance of Cu NLs@Co(OH)_2_ was evaluated in an H‐cell with 1 M KOH and 250 mM KNO_3_ as catholyte and 1 M KOH as anolyte. As shown in Figure 2a, the Cu NLs@Co(OH)_2_ exhibited a high current density of 2.15 A cm^−2^ at −1 V vs RHE, much higher than that of Cu NLs (1.75 A cm^−2^) and bare Cu foam (0.56 A cm^−2^). The selectivity toward NH_3_ for Cu NLs@Co(OH)_2_ and Cu NLs was further investigated. The Cu NLs@Co(OH)_2_ exhibited a high FEs over 90% within the potential window from −0.8 to −1 V vs RHE (Figure 2b), much higher than those of Cu NLs (<60%). Notably, the Cu NLs@Co(OH)_2_ showed a rapid NH_3_ yield rate of 2.02 × 10^−6^ mol s^−1^ cm^−2^, which is around two times the yield rate of Cu NLs (0.98 × 10^−6^ mol s^−1^ cm^−2^, Figure 2c). The NO_3_RR performance of Cu NLs@Co(OH)_2_ under different nitrate concentrations was further investigated. As shown in Figure 2d, the NO_3_RR activity of Cu NLs@Co(OH)_2_ increases under higher nitrate concentration with the highest current density of 2.35 A cm^−2^ at −1 V vs RHE in 1 M KOH with 1000 mM KNO_3_. In addition, the FEs toward NH_3_ are not compromised under high nitrate concentration, at −1.1 V vs RHE, with ∼100% and 96.82% for 500 mM and 1000 mM KNO_3,_ respectively (Figure 2e). As a result of both high current density and high FE, Cu NLs@Co(OH)_2_ achieves ultra‐high NH_3_ yield rate of 2.67 × 10^−6^ mol s^−1^ cm^−2^ with 500 mM KNO_3_ and 3.18 × 10^−6^ mol s^−1^ cm^−2^ with 1000 mM KNO_3_, respectively (Figure 2f). Moreover, Cu NLs@Co(OH)_2_ showed excellent stability with the FEs of ∼95% and NH_3_ yield rate of ∼2.0 × 10^−6^ mol s^−1^ cm^−2^ at −1 V vs RHE in 1 M KOH with 250 mM KNO_3_ during the measured 12 cycles (Figure 2g). The morphological and structural characterization of Cu NLs@Co(OH)_2_ was performed after cycling. As shown in Figure S6, the core‐shell nanosheet architecture of Cu NLs@Co(OH)_2_ was well‐maintained after the stability test. The post‐reaction XRD patterns display pronounced peaks of Co(OH)_2_, while the diffraction peaks of Cu_2_O disappeared under the applied reduction potential (Figure S7). Above results collectively demonstrated the excellent durability of the electrode in NO_3_RR. The superior NH_3_ synthesis performance of Cu NLs@Co(OH)_2_ is underscored by benchmarking against previously reported representative NO_3_RR electrocatalysts (Figure 2h and Table S2). NH_3_ yield rate of 3.18 × 10^−6^ mol s^−1^ cm^−2^ has been achieved, which surpasses most state‐of‐the‐art NO_3_RR catalysts and exceeds the U.S. Department of Energy's target for electrochemical ammonia synthesis device (9.7 × 10^−7^ mol s^−1^ cm^−2^) by over threefold [31]. To demonstrate the potential in wastewater treatment applications for Cu NLs@Co(OH)_2_, the NO_3_^−^ removal experiments in 1 M KOH with 100 mM KNO_3_ was performed. As shown in Figure S8, NO_3_^−^ is gradually consumed in the electroreduction accompanied by the generation of NH_3_. 97.7% of NO_3_^−^ was removed in 1 h, highlighting the promise of Cu NLs@Co(OH)_2_ for wastewater nitrate treatment and resource recovery.

### 2.3 NO_3_RR Mechanism Over Cu NLs@Co(OH)_2_

**FIGURE 3:**
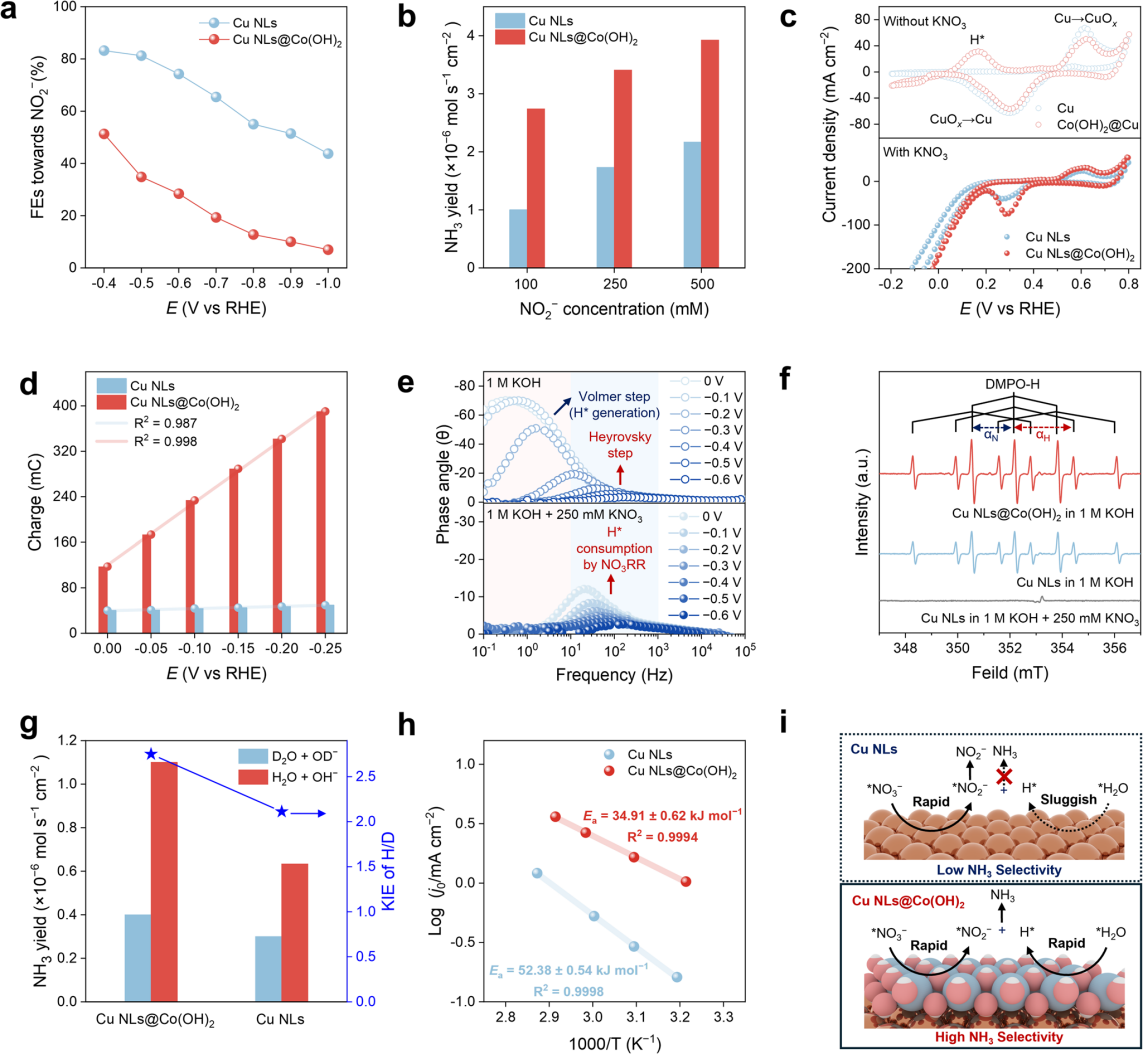
NO_3_RR mechanism investigation. (a) FEs toward NO_2_^−^ of Cu NLs and Cu NLs@Co(OH)_2_ in 1 M KOH with 250 mM NO_3_^−^. (b) NH_3_ yield rate of Cu NLs and Cu NLs@Co(OH)_2_ in 1 M KOH with varying NO_2_^−^ concentration. (c) cyclic voltammetry (CV) curves of Cu NLs and Cu NLs@Co(OH)_2_ in 1 M KOH with and without 250 mM NO_3_^−^. (d) Charge vs potential from pulse voltammetry of Cu NLs and Cu NLs@Co(OH)_2_. (e) Bode phase plots of Cu NLs@Co(OH)_2_ at varied potentials in 1 M KOH with and without 250 mM NO_3_^−^. (f) EPR spectra of Cu NLs and Cu NLs@Co(OH)_2_ in 1 M KOH and 1 M KOH with 250 mM NO_3_^−^. (g) KIE of H/D over Cu NLs and Cu NLs@Co(OH)_2_ in 1 M KOH/KOD with 250 mM NO_3_^−^. (h) Arrhenius plots of the HER exchange current densities on Cu NLs and Cu NLs@Co(OH)_2_ in 1 M KOH. (i) Schematic illustration of NO_3_RR mechanism over Cu NLs and Cu NLs@Co(OH)_2_.

To elucidate the origin of the improved NO_3_RR performance of the Cu NLs@Co(OH)_2_, the NO_3_RR mechanism over Cu NLs and Cu NLs@Co(OH)_2_ was further investigated. The NO_3_RR in alkaline media is a complex process with the transfer of eight electrons and nine protons involved (NO_3_^−^ + 6H_2_O + 8e^−^ → NH_3_ + 9OH^−^). Therefore, various nitrogen‐containing intermediates are produced in this process, such as: *NO_2_^−^, *NO, and *NH_2_OH [32, 33, 34]. Among them, NO_2_^−^ is a key intermediate in NO_3_RR over Cu‐based catalyst, and Cu plays a vital role in regulating NO_3_^−^/ NO_2_^−^ adsorption and N−O bond breaking [35, 36]. As shown in Figure 3a, NO_2_^−^ is the major product of NO_3_RR over Cu NLs in 1 M KOH with 250 mM KNO_3_, with the FE of 83.19% at −0.4 V vs RHE. In comparison, the FE toward NO_2_^−^ over Cu NLs@Co(OH)_2_ is 51.31% at −0.4 V vs RHE and only 6.97% at −1.0 V vs RHE, which strongly indicates the contribution of Co(OH)_2_ shell in subsequent conversion of NO_2_^−^ into NH_3_. In addition, the NH_3_ yield rates over Cu NLs@Co(OH)_2_ with KNO_2_ as the nitrogen source are significantly higher than those over Cu NLs, illustrating the hydrogenation of NO_2_^−^ into NH_3_ is a sluggish process over Cu NLs (Figure 3b). It has been widely reported that the NO_3_RR requires the tandem generation and consumption of adsorbed hydrogen (H^*^) by water splitting, which inspired us to investigate the H^*^ generation ability of both catalysts [37]. As shown in Figure S9, Cu NLs@Co(OH)_2_ exhibited better hydrogen evolution reaction (HER) activity with the current density of 0.83 A cm^−2^ at −0.8 V vs RHE, much higher than that of Cu NLs (0.43 A cm^−2^).

The dynamic generation and consumption of H^*^ was further monitored by various techniques. As shown in Figure 3c, the distinct oxidation peak at 0.15 V vs RHE in 1 M KOH is associated with the oxidation of generated H^*^ on Cu NLs@Co(OH)_2_ while no obvious H^*^ oxidation peak was observed for Cu NLs, indicating the weak H^*^ generation and storage ability of Cu NLs. After adding 250 mM KNO_3_ in the electrolyte, the H^*^ oxidation peak was completely suppressed, demonstrating that the in situ produced H^*^ was utilized for NO_3_RR effectively. The pulse voltammetry was applied with lower potential to store the H^*^ and higher potential to consume (Figure S10). The integral result of oxidation current (Figure S11) implies the amount of H^*^ generated on the electrode [38, 39]. As shown in Figure 3d, the H^*^ generation ability of Cu NLs@Co(OH)_2_ is ∼8 times that of Cu NLs. The operando electrochemical impedance spectra (EIS) were applied to monitor the generation of H^*^ on Cu NLs and Cu NLs@Co(OH)_2_. In the Bode plots of Cu@Co(OH)_2_, the intensity of the phase angle peak indicates the rate of charge transfer at the reaction interface (Figure 3e and Figure S12). The phase angle peak at low frequency area (0.1 to 10 Hz) indicated the Volmer step (H_2_O + M + e^−^ → M−H^*^ + OH^−^) of HER [40, 41, 42]. The phase angle peak at higher frequency area (10 to 100 Hz) indicated the consumption of H^*^. After adding KNO_3_ in the electrolyte, the peaks for Volmer step disappeared, indicating the consumption of H^*^ in NO_3_RR. As shown in Figure S13, the Volmer step peaks for Cu@Co(OH)_2_ shifted to lower angle, indicating more efficient water splitting process of Cu@Co(OH)_2_. To directly capture the H* during water splitting and NO_3_RR, Electron paramagnetic resonance (EPR) using 5,5‐dimethyl‐1‐pyrroline‐N‐oxide (DMPO) as the radical trapping reagent was also performed. As shown in Figure 3f, both electrodes exhibit the characteristic signal pattern of DMPO‐H adducts in 1 M KOH at −0.6 V vs RHE with an intensity ratio of ∼1:1:2:1:2:1:2:1:1 and calculated hyperfine coupling constants of *α*_N_ = 43.44 MHz and *α*_H_ = 63.25 MHz. Moreover, the signal intensity of DMPO‐H for Cu NLs@Co(OH)_2_ is strong than that of Cu NLs, demonstrating a better water splitting ability of Cu NLs@Co(OH)_2_ to generate H*. After adding 250 Mm KNO_3_ in the electrolyte, the signal for DMPO‐H vanished, indicating the involvement of H* in NO_3_RR. The kinetic isotope effect (KIE) analysis was applied to highlight the proton‐coupled electron transfer in NO_3_RR (Figure 3g). Compared with bare Cu NLs (KIE = 2.11), Cu NLs@Co(OH)_2_ exhibited a larger KIE (2.75), indicating that the rate‐determining step involves proton‐coupled electron transfer. The stronger isotope dependence suggests that Co(OH)_2_ facilitates water dissociation and enhances the supply of reactive H^*^, which directly accelerates the hydrogenation of NO_2_^−^ to NH_3_. Besides, the kinetics of H^*^ generation was also analyzed via the temperature dependent water splitting experiment (Figure S14). The activation energy (*E*a) of water splitting was obtained based on the correlation between the exchange current density (*j*_0_) and the temperature (Figure S15) [43, 44, 45, 46]. The *E*a of Cu NLs@Co(OH)_2_ was estimated to be 34.91  kJ  mol^−1^ (Figure 3h), significantly lower than that of Cu NLs (52.38 kJ  mol^−1^), revealing the excellent inherent H^*^ generation activity provided by Co(OH)_2_. Based on the investigation above, the enhanced NO_3_RR performance of the Cu NLs@Co(OH)_2_ was concluded in Figure 3i. On Cu NLs, the reduction of NO_3_^−^ to NO_2_^−^ occurs readily while the sluggish water dissociation severely limits H^*^ availability, preventing effective hydrogenation of NO_2_^−^ to NH_3_ and resulting in low NH_3_ selectivity. In sharp contrast, coating Cu NLs with an Co(OH)_2_ shell accelerates interfacial water activation and provides abundant H^*^, enabling rapid coupling between NO_2_^−^ and H^*^. The balanced supply of reactive nitrogen intermediates and hydrogen species allows the Cu NLs@ Co(OH)_2_ catalyst to achieve high NH_3_ selectivity.

### 2.4 FOR Performance of Cu NLs@Co(OH)_2_

**FIGURE 4:**
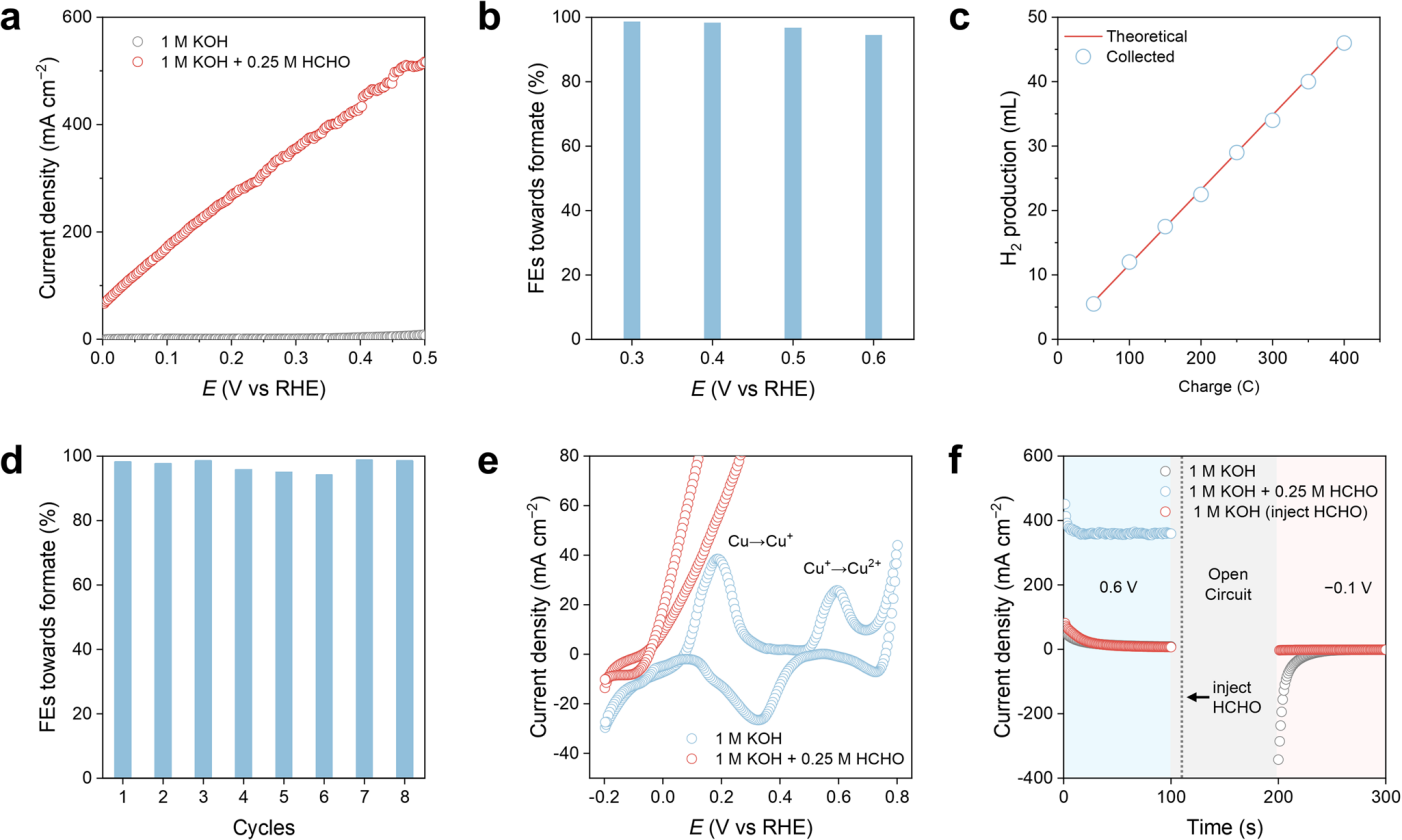
FOR performance of Cu NLs@Co(OH)_2_. (a) LSV curves of Cu NLs@Co(OH)_2_ in 1 M KOH with and without 250 mM HCHO. (b) FEs toward formate of Cu NLs@Co(OH)_2_ in 1 M KOH with 250 mM HCHO. (c) H_2_ production amount collected during FOR at 0.6 V vs RHE in 1 M KOH with 250 mM HCHO. (d) FEs toward formate during 8 continuous cycles for Cu NLs@Co(OH)_2_ at 0.6 V vs RHE in 1 M KOH with 250 mM HCHO. (e) CV curves of Cu NLs@Co(OH)_2_ in 1 M KOH with and without 250 mM HCHO. (f) Multi‐potential step curves for Cu NLs@Co(OH)_2_ in 1 M KOH with and without 250 mM HCHO.

To achieve co‐production of NH_3_ and electricity, FOR was selected as the anodic reaction to couple with cathodic NO_3_RR. As shown in Figure 4a, Cu NLs@Co(OH)_2_ also showed excellent FOR activity with a high current density of 517.03 mA cm^−2^ at 0.5 V vs RHE in 1 M KOH with 250 mM HCHO, much higher than that in 1 M KOH without 250 mM HCHO (8.22 mA cm^−2^). Remarkably, the catalyst achieves high FEs over 90% for formate over a wide potential range of 0.30 V to 0.60 V vs RHE (Figure 4b). Besides, the volume of H_2_ collected at anode during FOR showed excellent stoichiometric agreement with the theoretical H_2_ volumes (Figure 4c), verifying that FOR on Cu NLs@Co(OH)_2_ follows a one‐electron pathway that co‐produces H_2_ and formate (Figure S16). In addition, Cu NLs@Co(OH)_2_ showed no drop in FEs toward formate at 0.6 V vs RHE in 1 M KOH with 250 mM HCHO during the measured 8 cycles (Figure 4d), demonstrating its excellent stability.

The FOR mechanism was first investigated by cyclic voltammetry (CV) in 1 M KOH electrolyte. As shown in Figure 4e, Cu NLs@Co(OH)_2_ electrode displays 2 distinct redox peaks for Cu//Cu^+^ and Cu^+^//Cu^2+^. Upon addition of HCHO, the anodic current rises dramatically while these redox features vanish, suggesting that the Cu^+^ and Cu^2+^ intermediates are no longer accumulated but instead immediately reduced by HCHO. This disappearance of Cu redox peaks confirms that both Cu^+^ and Cu^2+^ actively participate in the FOR. Furthermore, a multi‐potential step chronoamperometric measurement was carried out. As shown in Figure 4f, the positive current observed at 0.60 V vs RHE can be ascribed to the electrooxidation of the electrode in 1 M KOH. After open circuit measurement, a potential of −0.1 V vs RHE was applied to Cu NLs@Co(OH)_2_ electrode, and the negative current indicates the electroreduction of Cu^2+^. In 1 M KOH with 0.25 M HCHO, the oxidation current density was greatly enhanced to ∼360 mA cm^−2^, indicating the oxidation of HCHO. However, no reduction current was observed at −0.1 V vs RHE. It is speculated that the Cu^2+^ cannot be accumulated on the surface of Cu NLs@Co(OH)_2_ in the presence of HCHO. To verify this hypothesis, we carried out a third control experiment: First, Cu^2+^ was accumulated on the Cu NLs@Co(OH)_2_ electrode (0–100 s: 1 M KOH). Then, 0.25 M HCHO was injected into 1 M KOH during the open circuit state (100–200 s). After this, the reduction current completely disappeared at −0.1 V vs RHE (200–300s: 1 M KOH with 0.25 M HCHO), indicating that the accumulated Cu^2+^ was completely reduced into Cu by HCHO spontaneously accompanied by the co‐generation of H_2_ and formate (Figure S17).

### 2.5 Symmetric Hydrogen, Ammonia and Formate Co‐Generation Fuel Cell

**FIGURE 5:**
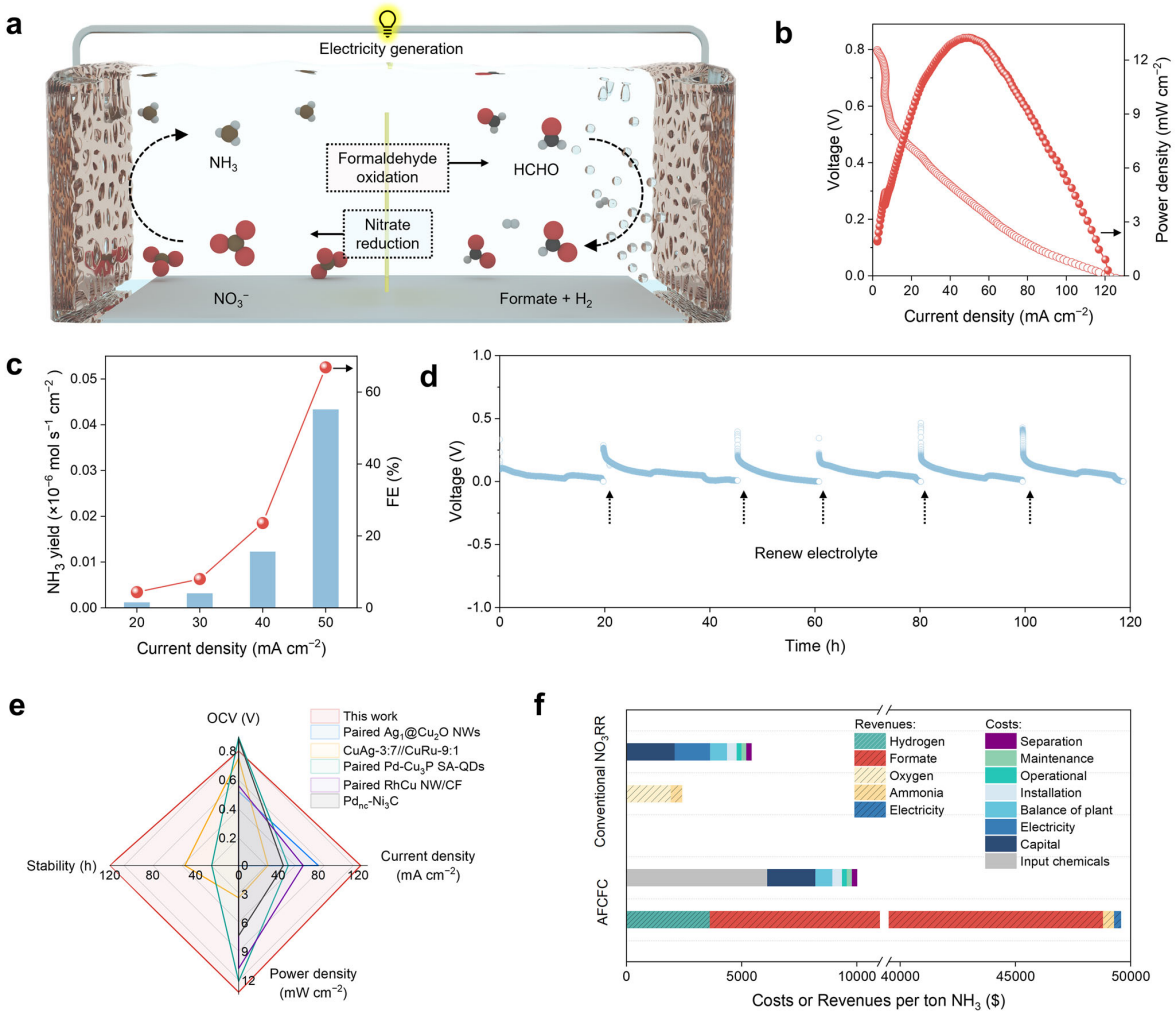
NH_3_ synthesis via AFCFC. (a) Schematic illustration of AFCFC. (b) Performance of AFCFC. (c) FEs toward NH_3_ and NH_3_ yield rates of AFCFC. (d) Discharge stability of AFCFC at 20 mA cm^−2^. (e) Comparison between the performance of the AFCFC and those of previously reported ammonia‐production fuel cells. (f) Breakdown of costs and revenues associated with NH_3_ production via the AFCFC compared with a conventional NO_3_RR process.

Based on the superior performance of Cu NLs@Co(OH)_2_ in both NO_3_RR and FOR, a symmetric ammonia formate co‐production fuel cell (AFCFC) with Cu NLs@Co(OH)_2_ serving as both anode and cathode was designed to enable unassisted electrochemical NH_3_ synthesis and the co‐generation of formate, H_2_ and electricity (Figure 5a). The OCV of this AFCFC was first evaluated. As shown in Figure S18, there is no OCV when the anolyte and catholyte are both 1 M KOH. After adding 0.25 M KNO_3_ at cathode, the OCV still remained at ∼0 V. Interestingly, the OCV increased rapidly to ∼0.80 V after adding 0.5 M HCHO at anode, highlighting the necessity to couple NO_3_RR with FOR to generate electricity. In addition, the OCV of the AFCFC barely changed during the measured 60 mins (Figure S19). The excellent activity of Cu NLs@Co(OH)_2_ in both NO_3_RR and FOR enabled the AFCFC to achieve a maximum discharge current density of 122.93 mA cm^−2^ and a peak power density of 13.24 mW cm^−2^ (Figure 5b).

The NH_3_ and formate synthesized via AFCFC under different discharge current density was further detected. As shown in Figure 5c and Figures S20 and S21, the NH_3_ yield rates gradually enhanced with the increase of discharge current density. The AFCFC achieved a yield rate of 0.043 × 10^−6^ mol s^−1^ cm^−2^ and FE of 66.90% for NH_3_ generation. Meanwhile, formate can be obtained at anode with and a yield rate of 0.53 × 10^−6^ mol s^−1^ cm^−2^ and FE of 98.10%, further confirming the potential application of the electrochemical synthesis along with the electricity generation. Furthermore, the AFCFC delivers stable power output for over 120 h with a high discharge current density of 20 mA cm^−2^ (Figure 5d). The superior performance of the AFCFC is further highlighted by comparison with previous work on unassisted electrochemical NH_3_ synthesis system. The AFCFC exhibited all‐round advantages in OCV, discharge current density, power density and stability compared to previous reported systems (Figure 5e, Table S3).

The technoeconomic analysis (TEA) was further introduced to evaluate the cost and revenues of the NH_3_ synthesis via AFCFC (Figure 5f and Note S1). The conventional NO_3_RR coupled with OER requires high energy input, resulting in a high electricity cost. In addition, the O_2_ generated at the anode has relatively low value, which leads to a poor profit of conventional NO_3_RR. In comparison, the AFCFC can be operated without power input, instead of generating electricity. Moreover, the value‐added formate and H_2_ produced at anode contributed to a significant revenue, achieving an estimated profit of $39575.43 for per ton of produced NH_3_. This corresponds to a negative net production cost for electrochemical ammonia synthesis under the assumptions used here.

## 3 Conclusion

In conclusion, we developed a symmetric cogeneration fuel cell that integrates value‐added chemical synthesis with electricity generation by coupling one‐electron formaldehyde oxidation reaction with the nitrate reduction. A bifunctional Cu NLs@Co(OH)_2_ catalyst was developed as both the cathode for NO_3_RR and the anode for FOR. At −1.0 V vs RHE, Cu NLs@Co(OH)_2_ delivers an industrially relevant current density of 2.35 A cm^−2^ together with a high ammonia Faradaic efficiency of 96.38%. Mechanistic analysis reveals that the outstanding NO_3_RR performance originates from a balanced supply of reactive nitrogen intermediates and active hydrogen species enabled by the Cu‐Co(OH)_2_ interfacial architecture. By coupling NO_3_RR with FOR, the bifunctional catalyst enables a self‐powered formaldehyde‐nitrate device that drives the unassisted co‐synthesis of hydrogen, ammonia and formate. Operating at 35°C, the symmetric cogeneration fuel cell achieves an OCV of 0.80 V, a peak power density of 13.24 mW cm^−2^, and stable operation over 120 h. This work establishes a simple yet effective strategy for integrating value‐added chemical synthesis with electricity generation in a single electrochemical platform and offers a generalizable framework that can be extended to other paired electrochemical synthesis systems.

## 4 Methods

### 4.1 Preparation of Cu NLs@Co(OH)_2_

A two‐step electrodeposition method was applied to the preparation of Cu NLs@Co(OH)_2_. First, the copper foam was cut into 1 × 1.5 cm^2^ and ultrasonically cleaned in ethanol, 5% HCl solution and deionized water for 15 min each to remove the grease and oxide layer on the surface of copper foam. Then, the cleaned copper foam, the carbon rod and Ag/AgCl electrodes was used as working electrode, counter electrode and reference electrode, respectively. The electrodeposition electrolyte was prepared by dissolving 1.3 g CuSO_4_·5H_2_O and 3.9 mL H_2_SO_4_ (18 M) in 32 mL deionized water. Cu NLs was obtained by electrodeposition at −1.0 V for 180 s. In the second step electrodeposition, the prepared Cu NLs, the carbon rod and Ag/AgCl electrodes was used as working electrode, counter electrode and reference electrode, respectively. The electrodeposition electrolyte was 0.15 M Co(NO_3_)_2_ solution. Cu NLs@Co(OH)_2_ was obtained by electrodeposition at −1.0 V for 300 s. All the electrodes were cleaned by deionized water after electrodeposition and dried at 60°C overnight.

### 4.2 Structural Characterizations

The X‐ray diffraction (XRD) was carried out on a third‐generation Malvern Panalytical Empyrean equipped with multicore (iCore/dCore) optics and a Pixcel3D detector operating in 1D scanning mode with a Cu *K*α radiation (1.5419 Å) to identify the crystalline phases present in the samples. The diffraction scans were collected over a 2*θ* range from 10° to 90° at a step size of 5° min^−1^ were analyzed using the Malvern Panalytical Highscore Plus 4.9 software and the latest ICDD PDF‐4+ database.

Scanning electron microscopy (SEM) was carried out on a Zeiss SUPRA 55‐VP scanning microscope. Annular dark field (ADF) and bright field (BF) scanning transmission electron microscopy (STEM) imaging and EDX elemental mapping were carried out on a double aberration‐corrected JEOL ARM200F TEM, operated at 200 kV, equipped with a 100 mm^2^ Oxford Instruments windowless EDX detector.

The X‐ray photoelectron spectroscopy (XPS) data were collected at the Photoemission Research Technology Platform, University of Warwick. The samples investigated in this study were attached to electrically‐conductive carbon tape, mounted on to a sample bar with a layer of filter paper between the samples and the sample bar to ensure electrical isolation and hence mitigate differential charging, before being loaded into a Kratos Axis Ultra DLD spectrometer which possesses a base pressure below 1 × 10^−10^ mbar. XPS measurements were performed in the main analysis chamber, with the sample being illuminated using a monochromated Al Kα X‐ray source (h*ν* = 1486.7 eV). The measurements were conducted at room temperature and at a take‐off angle of 90° with respect to the surface parallel. The core level spectra were recorded using a pass energy of 20 eV (resolution approx. 0.4 eV), from an analysis area of 300 × 700 µm. The work function and binding energy scale of the spectrometer were calibrated using the Fermi edge and 3*d*_5/2_ peak recorded from a polycrystalline Ag sample prior to the commencement of the experiments. To prevent surface charging the surface was flooded with a beam of low energy electrons from a charge neutralizer throughout the experiment and this necessitated recalibration of the binding energy scale. To achieve this, the C−C/C−H component in the C 1s spectrum was referenced to 285.0 eV. The data were analyzed in the CasaXPS package using Shirley backgrounds and mixed Gaussian‐Lorentzian (Voigt) line shapes. For compositional analysis, the analyzer transmission function has been determined using clean metallic foils to determine the detection efficiency across the full binding energy range.

### 4.3 EPR Spectroscopy

EPR spectroscopy was performed on a continuous wave X‐band spectrometer (Bruker, EMX) fitted with a cylindrical cavity resonator. Typically, the electrochemical reaction was conducted at −0.6 V vs RHE for a duration of 2 min. Subsequently, 20 uL of the DMPO was added into 4 mL electrolyte before capturing the DMPO‐H signals. Solutions were placed in quartz EPR tubes of 1 mm ID (Wilmad quartz (CFQ)). For all measurements, the following optimized spectrometer parameters were used: microwave power of 12 mW at a nominal 9.884 GHz frequency; central magnetic field, 352 mT; sweep width, 20 mT; and modulation amplitude, 0.1 mT. All spectra reported are an average of 4 scans. All EPR data was fitted with simulated spin adducts using the MATLAB package EasySpin (Version 6.0.12) [47].

### 4.4 Electrochemical Measurements

Electrochemical measurements were performed on a Solartron 1287 A electrochemical interface controlled by electrochemical software Corr‐Ware/CorrView. The EIS data of the fuel cell was collected by the Solartron 1260 A Electrochemical Station at a frequency range of 1 MHz to 0.01 Hz. All electrolyte was thoroughly purged with Ar to eliminate dissolved N_2_ and other potential nitrogen contaminants. The measured potentials vs the Ag/AgCl reference electrode were converted to the reversible hydrogen electrode (RHE) scale via the Nernst equation:

(3) $$E_{RHE}=E_{\mathrm{Ag}/\mathrm{AgCl}}+0.059\;pH+0.197$$

### 4.5 NO3RR Products Measurements

The produced NH_3_ was quantitatively detected by the modified indophenol blue method. Typically, 5 g salicylate, 5 g potassium sodium tartrate and 2 g NaOH were dissolved in 100 mL deionized water to form solution A. 1 g sodium nitroprusside was dissolved in 100 mL deionized water to form solution B. 4 mL 13% wt. NaClO solution and 3 g NaOH was dissolved in 100 mL deionized water to form solution C. 1 mL solution A, 100 µL solution B, and 100 µL solution C solution were mixed uniformity with 10 mL diluted electrolyte and then stood for 2 h. The absorption intensity at a wavelength of 661 nm was recorded on a UV–vis absorption spectrometer (Shimadzu 2600, Japan). The concentration‐absorbance calibration curve was calibrated using a series of standard NH_4_Cl solutions (Figure S22). The concentration of NO_3_^−^ and NO_2_^−^ was determined by ion chromatography (Metrohm 883 IC, with Metrosep C4−250/4.0 column for cations and ICSep AN2 column for anions) using an electrical conductivity detector. The concentration‐absorbance calibration curve was calibrated using a series of standard KNO_2_+KNO_3_+KOH solutions (Figure S23).

The Faradaic efficiency (FE) toward NH_3_ can be calculated by:

(4) $$\mathrm{FE}=\frac{8\times N\left(NH_{3}\right)\times F}{Q}$$

where 8 is eight electrons are required to convert one NO_3_^−^ molecule to one NH_3_ molecules, *N(*NH_3_) is the total amount (in units of moles) of NH_3_ obtained from chronoamperometry test, *F* is the Faraday constant (*F* = 96485 C mol^−1^), *Q* is the total charge passed through the electrochemical cell.

The Faradaic efficiency (FE) toward NO_2_^−^ can be calculated by

(5) $$\mathrm{FE}=\frac{2\times N\left(NO_{2}^{-}\right)\times F}{Q}$$

where 2 is two electrons are required to convert one NO_3_^−^ molecule to one NO_2_^−^ molecules, *N(*NO_2_^−^) is the total amount (in units of moles) of NO_2_^−^ obtained from chronoamperometry test, *F* is the Faraday constant (*F* = 96485 C mol^−1^), *Q* is the total charge passed through the electrochemical cell.

### 4.6 FOR Products Measurements

The produced formate was quantitatively detected by ion chromatography (Metrohm 883 IC, with Metrosep C4−250/4.0 column for cations and ICSep AN2 column for anions) using an electrical conductivity detector. The concentration‐absorbance calibration curve was calibrated using a series of standard HCOOK+KOH solutions (Figure S24).

The Faradaic efficiency (FE) toward formate can be calculated by

(6) $$\mathrm{FE}=\frac{1\times N\left(\mathrm{form}\mathrm{ate}\right)\times F}{Q}$$

where 1 is one electron required to convert one HCHO molecule to one formate molecule, *N(*formate) is the total amount (in units of moles) of formate obtained from chronoamperometry test, *F* is the Faraday constant (*F* = 96485 C mol^−1^), *Q* is the total charge passed through the electrochemical cell.

### 4.7 Ammonia Formate Co‐Production Fuel Cell

AFCFC was assembled with a working electrode area of 1 cm^−2^ using Cu NLs@Co(OH)_2_ as both cathode and anode for NO_3_RR with FOR (Figure S25). The membrane is the home made PAP‐TP85 anion exchange membrane (Figure S26) [48]. The electrolyte was circulated through the low chamber at a flow rate of 100 mL min^−1^ and maintained at a temperature of 35°C for tests at various current. The polarization curves and power density curves were measured through a on a Solartron 1470E CellT est System.

## Author Contributions

**Shanwen Tao**: conceived and supervised the project. **Shanwen Tao** and **Yingjie Song**: designed the experiments. **Yingjie Song**: performed the experiments except for STEM measurement, and XPS measurement, **Peimiao Zou**: helped with SEM experiments, **Qi Zhang**: helped with ammonia detection, **Yisong Han**: helped with STEM measurements and analysis. **Christopher Waldron** and **Marc Walker**: collected the XPS data and helped with analysis. **Ben G. Breeze**: helped with EPR measurements. **Shanwen Tao** and **Yingjie Song**: analysed the data and wrote the manuscript. All authors commented on the manuscript.

## Funding

This work was supported by the EPSRC (Grant No. EP/X038963/1); Hartnoll Centre, University of Warwick.

## Conflicts of Interest

The authors declare no conflicts of interest.

## Supporting information




**Supporting File**: advs75826‐sup‐0001‐SuppMat.docx.

## Data Availability

The data that support the findings of this study are available on request from the corresponding author. The data are not publicly available due to privacy or ethical restrictions.
