# Supplementary material for: A Symmetric Cogeneration Fuel Cell for Coupled Production of Hydrogen, Ammonia and Formate
Source: Adv Sci (Weinh). 2026 May 25:e75826. Online ahead of print. doi: 10.1002/advs.75826 (PMC13335825; doi:10.1002/advs.75826)
Supplement: Supplementary file 1 — Supporting File: advs75826‐sup‐0001‐SuppMat.docx. [file ADVS-9999-e75826-s001.docx]

Supporting Information

A Symmetric Cogeneration Fuel Cell for Coupled Production of Hydrogen, Ammonia and Formate

Yingjie Song, Peimiao Zou, Renhang Wang, Qi Zhang, Yisong Han, Christopher Waldron, Marc Walker, Ben G. Breeze and Shanwen Tao^*^

**Table of Contents**

[**Supplementary Notes** 4](#_Toc228957061)

[**Note S1: Techno-economic analysis** 4](#_Toc228957062)

[**Note S2: Preparation of PAP-TP-85 membrane** 7](#_Toc228957063)

[**Figures** 8](#_Toc228957064)

[**Figure S1. Schematic diagrams for the unassisted electrochemical NH_3_ synthesis.** 8](#_Toc228957065)

[**Figure S2. ADF-STEM images of Cu NLs@Co(OH)_2_.** 8](#_Toc228957066)

[**Figure S3. ADF-STEM image and corresponding map results of Cu NLs@Co(OH)_2_.** 9](#_Toc228957067)

[**Figure S4. X-ray diffraction spectroscopy of Cu NLs and Cu NLs@Co(OH)_2_.** 10](#_Toc228957068)

[**Figure S5. High-resolution O 1*s* XPS spectra of Cu NLs and Cu NLs@Co(OH)_2_.** 10](#_Toc228957069)

[**Figure S6. SEM images of Cu NLs@Co(OH)_2_ post stability test.** 11](#_Toc228957070)

[**Figure S7. X-ray diffraction spectroscopy of Cu NLs@Co(OH)_2_ post stability test.** 11](#_Toc228957071)

[**Figure S8. The time-dependent concentration of NO_3_^−^, NO_2_^−^, and NH_3_ at −1.0 V vs RHE in 1 M KOH + 0.1 M KNO_3_. for Cu NLs@Co(OH)_2_.** 12](#_Toc228957072)

[**Figure S9. HER performance of Cu NLs and Cu NLs@Co(OH)_2_.** 12](#_Toc228957073)

[**Figure S10. The potential change curves of pulse voltammetry.** 13](#_Toc228957074)

[**Figure S11. The current change curve of pulse voltammetry.** 14](#_Toc228957075)

[**Figure S12. Bode phase plots of Cu NLs at varied potentials in 1 M KOH with and without 250 mM NO_3_^−^.** 15](#_Toc228957076)

[**Figure S13. Bode phase plots of Cu NLs and Cu NLs@Co(OH)_2_ in 1 M KOH.** 16](#_Toc228957077)

[**Figure S14. Temperature dependent HER performance of Cu NLs and Cu NLs@Co(OH)_2_.** 17](#_Toc228957078)

[**Figure S15. Tafel plots of Cu NLs and Cu NLs@Co(OH)_2_.** 18](#_Toc228957079)

[**Figure S16. Schematic diagrams for the formaldehyde oxidation pathways.** 19](#_Toc228957080)

[**Figure S17. Formaldehyde oxidation reaction mechanism over Cu NLs@Co(OH)_2_.** 19](#_Toc228957081)

[**Figure S18. OCV changes curve of AFCFC.** 20](#_Toc228957082)

[**Figure S19. OCV stability curve of AFCFC.** 20](#_Toc228957083)

[**Figure S20. Discharge curve of AFCFC with different current density.** 21](#_Toc228957084)

[**Figure S21. FEs towards formate and formate yield rates of AFCFC.** 21](#_Toc228957085)

[**Figure S22. NH_3_ quantification using UV-vis absorption spectroscopy.** 22](#_Toc228957086)

[**Figure S23. NO_2_^−^ and NO_3_^−^ quantification using ion chromatography.** 22](#_Toc228957087)

[**Figure S24. formate quantification using ion chromatography.** 23](#_Toc228957088)

[**Figure S25. Schematic diagram and digital photo for the AFCFC device.** 24](#_Toc228957089)

[**Figure S26. Digital photos of home-made PAP-TP-85-N membrane.** 25](#_Toc228957090)

[**Figure S27. Model of the techno-economic analysis of electrochemical ammonia production.** 26](#_Toc228957091)

[**Figure S28. The polarisation curve of NO_3_RR coupled with OER.** 27](#_Toc228957092)

[**Supplementary Tables** 28](#_Toc228957093)

[**Table S1. XPS composition reports of Cu NLs and Cu NLs@Co(OH)_2_.** 28](#_Toc228957094)

[**Table S2. Comparison of the NO_3_RR performance of Cu NLs@Co(OH)_2_ with other reported electrocatalysts in recent years.** 29](#_Toc228957095)

[**Table S3. Comparison of the performance of AFCFC with other reported unassisted NH_3_ synthesis system in recent years.** 30](#_Toc228957096)

[**Table S4.** **Price^a)^ of input chemicals and products.** 31](#_Toc228957097)

[**References** 32](#_Toc228957098)

**Supplementary Notes**

**Note S1: Techno-economic analysis**

The techno-economic analysis was carried out using a modified model to calculate the total plant gate levelized cost of production with units of US$ per tonne of ammonia. As shown in Figure S27, the costs are separated into 2 components, namely the capital costs and the operating costs. Capital costs is assumed to consist of the electrolyser and catalysta cost. Operating costs can be broken down into 7 components: electricity costs, maintenance costs, product separation costs, installation costs, balance of plant, input chemical costs and other operational costs.

A plant is assumed to produce 1 ton of ammonia per day and the plant lifetime is assumed to be 30 years. Capacity factor is the fraction of time the plant is expected to be operational on any given day, and this is assumed to be 0.8. This means the plant will be operational 19.2 hours a day. The Faradaic efficiency is assumed to be 60% under the current density (*i*) of 50 mA cm^−2^. To calculate capital costs, we assume a cost of $10000 per m^2^ of electrolyzer and the catalyst costs are assumed to be 5% of the electrolyser costs^[1-2]^.

The maintenance costs, product separation costs and other operational costs are assumed to be 10% of the capital costs. The maintenance costs, product separation costs and other operational costs are assumed to be 10% of the capital costs. The installation costs is assumed to be 20% of the capital costs. The balance of plant costs is assumed to be 35% of the capital costs.

The charge required to produce per ton ammonia (*Q*) = $\frac{n\left( {NH}_{3} \right)\times N\times F}{FE}$

= $\frac{5.87\times{10}^{4} mol\times8\times96485 C/mol}{0.6}$= 7.55×10^10^ C

Where N is 8 electrons are required to convert one KNO_3_ molecule to NH_3_, *n(*NH_3_*)* is the total amount (in units of moles) of NH_3_, *F* is the Faraday constant (*F* = 96485 C mol^−1^), FE is the Faradaic efficiency.

The current required to sustain the process (*I*) = *Q*/operational time

= 7.55×10^10^/(19.2 ×3600) = 1.09×10^6^ A.

Electrolyzer costs = Area of the electrolyzer × $10000

= *I*/the operation current density (*i*) × $10000

=1.09×10^6^ /0.05/10000×10000= 2.19×10^7^ $

Catalyst costs = Electrolyzer costs×5%

= 1.46×10^7^×0.05=1.09×10^6^ $

The capital costs = (Electrolyzer costs+Catalyst costs)/plant lifetime

= (2.19×10^7^+1.09×10^6^)/(30×365) = **2099.54 $** per ton ammonia

The maintenance costs = The capital costs×10%

= 2099.54×0.1=209.95 $

The product separation costs = The capital costs×10%

= 2099.54×0.1=209.95 $

Other operational costs = The capital costs×10%

= 2099.54×0.1=209.95 $

The installation costs = The capital costs×20%

= 2099.54×0.2=419.91 $

The balance of plant costs = The capital costs×35%

= 1399.82×0.35=734.84 $

The electricity price is assumed to be 5 cents/kWh. The electricity costs of per ton ammonia = *P*×0.05 = *I*×*E*×time*0.05. Where *P* is power required to sustain the process and *I* is the current required to sustain the process. *E* is the cell voltage, which is 1.47 V for conventional NO_3_RR cell (Figure S28) and −0.27 V for ammonia formate co-production fuel cell (AFCFC).

Therefore, the electricity costs for the conventional NO_3_RR cell per day (per ton ammonia) = 1.09×10^6^×1.47×0.05×19.2/1000 $ = 1541.78 $. The electricity costs for the AFCFC per day (per ton ammonia) = 1.09×10^6^×−0.27×0.05×19.2/1000 $ = −283.18$.

The mass of formaldehyde required = (mass of ammonia×8×molecular mass of formaldehyde)/ (molecular mass of ammonia×8) = 9.41 ton.

The input chemicals costs = mass of formaldehyde required × price of formaldehyde

= 9.41×650=6116.5 $

The operating costs for conventional NO_3_RR cell = electricity costs+maintenance costs+ product separation costs+installation costs+balance of plant+input chemical costs+other operational costs = 209.95×3+419.91+734.84+1541.78 = **3326.38 $**.

The operating costs for AFCFC cell = electricity costs+maintenance costs+ product separation costs+installation costs+balance of plant+input chemical costs+other operational costs = 209.95×3+419.91+734.84+0+6116.5 = **7901.1 $**.

The total costs for conventional NO_3_RR cell = capital costs +operating costs

= 2099.54+3326.38 = **5425.92 $** per ton ammonia

The total costs for AFCFC cell = capital costs +operating costs

= 2099.54+7901.1 = **10000.64 $** per ton ammonia

The total revenues can be calculated based on the market price of products (Table S1) from both anode and cathode.

For conventional NO_3_RR cell, the mass of produced oxygen at anode = (mass of ammonia × molecular mass of oxygen×8)/(molecular mass of ammonia×4) = 3.76 ton.

For AFCFC, the mass of produced formate at anode = (mass of ammonia×8×molecular mass of potassium formate)/(molecular mass of ammonia×1) = 39.53 ton.

The mass of produced hydrogen at anode = (mass of ammonia×8×molecular mass of hydrogen)/(molecular mass of ammonia×1) = 0.94 ton.

For conventional NO_3_RR cell, the total revenues = price of ammonia × mass of ammonia + price of oxygen × mass of oxygen

= 477×1+242×8 = **2413 $**

For AFCFC, the total revenues = price of ammonia × mass of ammonia + price of formate × mass of formate + price of hydrogen × mass of hydrogen+ electricity

= 477×1+1143×39.53+3865×0.94+283.18= **49576.07 $**

The price of chemicals and electricity was shown in Table S4.

**Note S2: Preparation of PAP-TP-85 membrane**

**Synthesis of PAP-TP-85-N**

A typical synthesis procedure of PAP-TP-85-N is as follows: to a 250 ml three-necked flask equipped with an overhead mechanical stirrer, *N*-methyl-4-piperidone (8.6239 g, 76.21 mmol), 2,2,2-trifluoroacetophenone (2.3419 g, 13.45 mmol) and *p*-terphenyl (20.6487 g, 89.66 mmol) were dissolved into methylene chloride (75 ml). TFA (6 ml) and TFSA (75 ml) were then added dropwise slowly at 0 °C. Thereafter, the reaction continued at this temperature for 24 h. The resulting viscous, dark blue solution was poured slowly into an aqueous solution of ethanol. The white fibrous solid was filtered, washed with water and immersed in 1 M K_2_CO_3_ at 50 °C for 12 h. Finally, the white fibrous product was filtered, washed with water and dried completely at 60 °C under vacuum.

**Synthesis of PAP-TP-85**

A typical synthesis procedure of PAP-TP-85 is as follows: to a 50 ml one-necked flask equipped with a magnetic bar, PAP-TP-85-N (1.0 g) was suspended in DMSO (20 ml). Methyl iodide (1 ml) was added quickly. The solution was stirred for more than 12 h at room temperature. The resulting viscous, yellow solution was added dropwise into ether. The yellow solid was filtered, washed with ether and dried completely at 60 °C under vacuum.

**Preparation of PAP membrane**

Membrane was prepared by dissolving the PAP-TP polymer (1.0 g) in DMSO (30 ml) and casting on a clean glass plate at 120 °C for 2 h. The membrane (in iodide form) was peeled off from the glass plate in contact with deionized water. Membrane in chloride ion form (0.5 g) was obtained by ion exchange in 1 M KCl solution at 80 °C for more than five times with each time taking 2 h, followed by washing and immersion in deionized water for 48 h to remove residual KCl. Membrane in hydroxide form (0.5 g) was obtained by ion exchange in 1 M KOH solution at 80 °C for more than five times with each time taking 2 h, followed by washing and immersion in deionized water under N_2_ (to avoid contact with CO_2_ and the formation of carbonate).

**Figures**


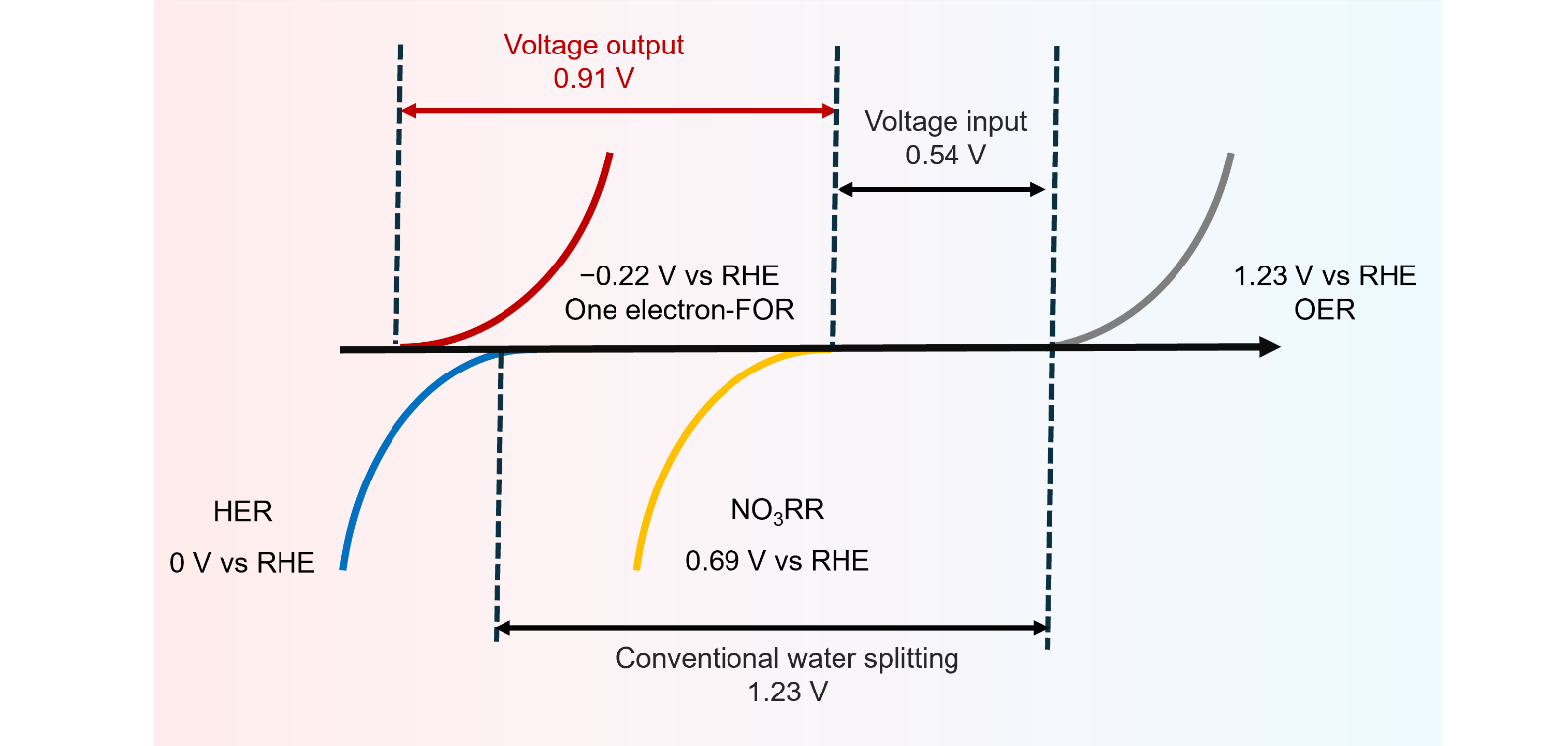


**Figure S1. Schematic diagrams for the unassisted electrochemical NH_3_ synthesis.**


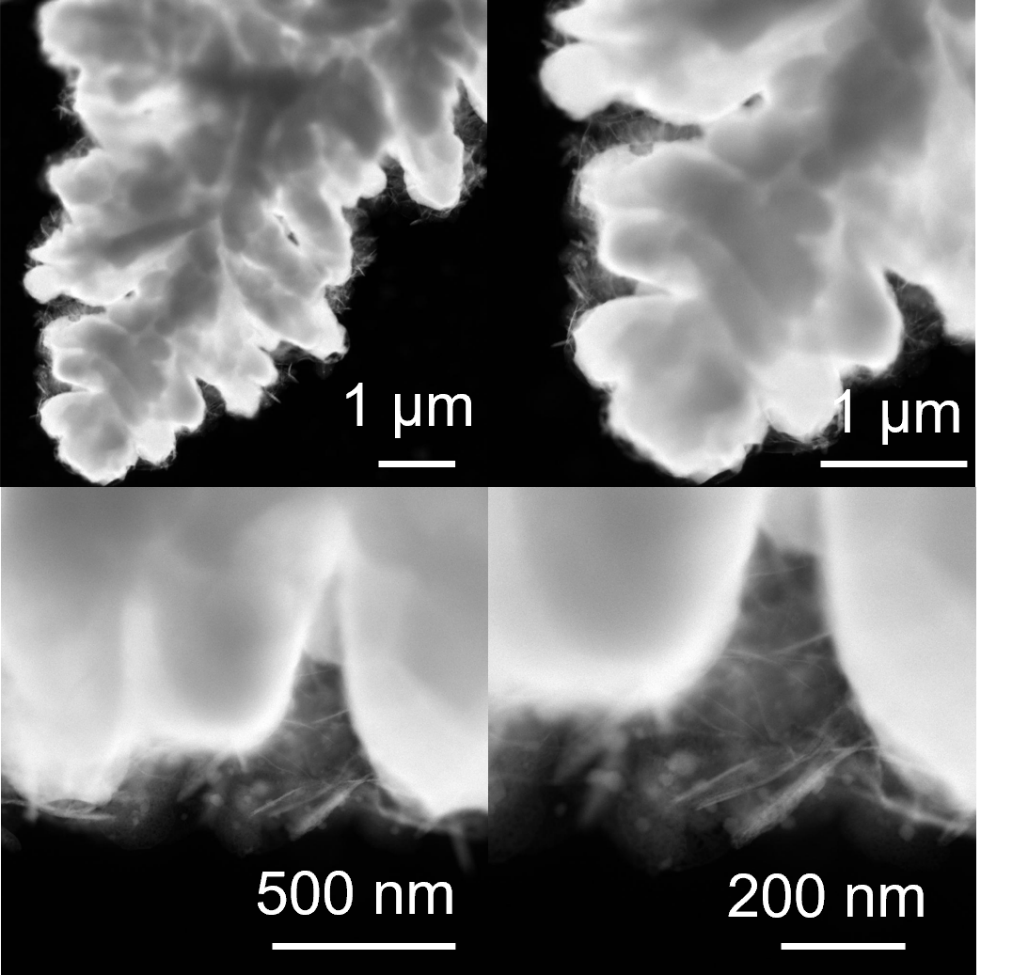


**Figure S2. ADF-STEM images of Cu NLs@Co(OH)_2_.**


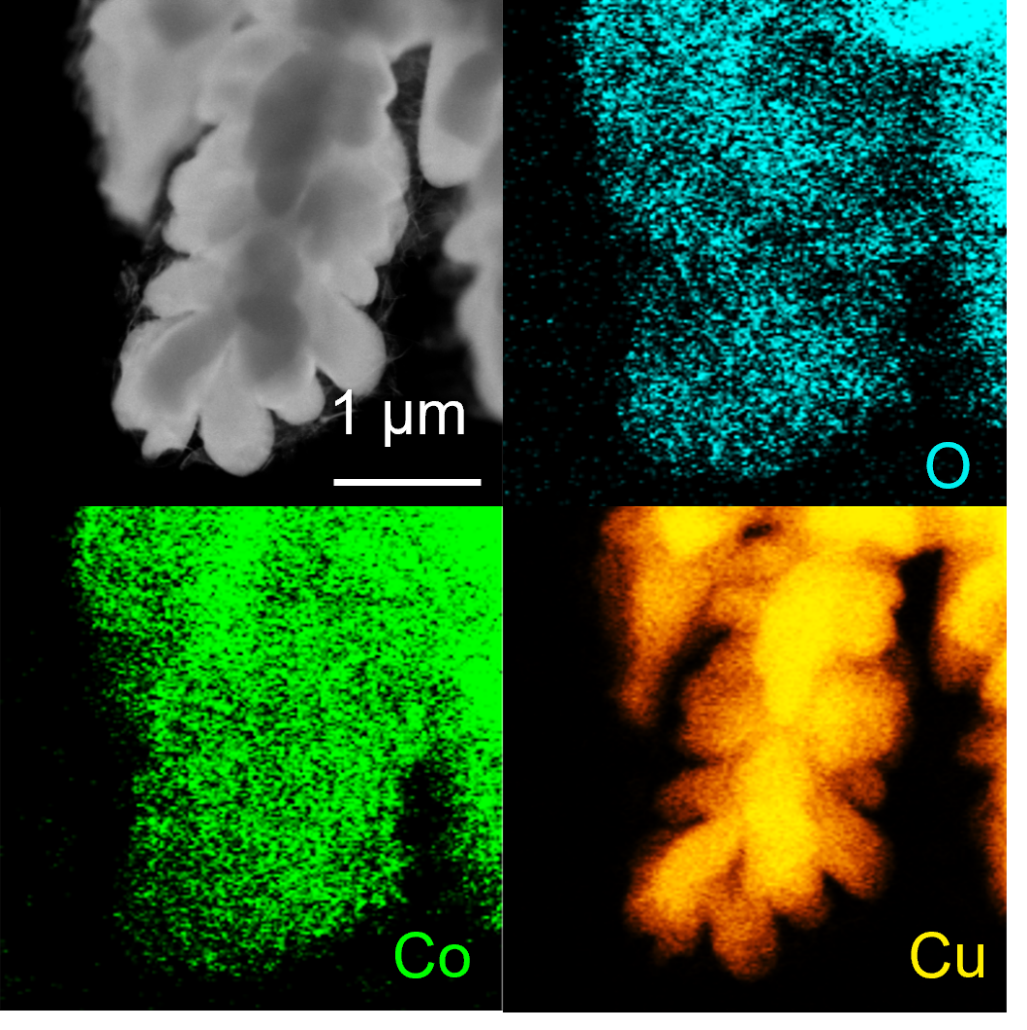


**Figure S3. ADF-STEM image and corresponding map results of Cu NLs@Co(OH)_2_.**


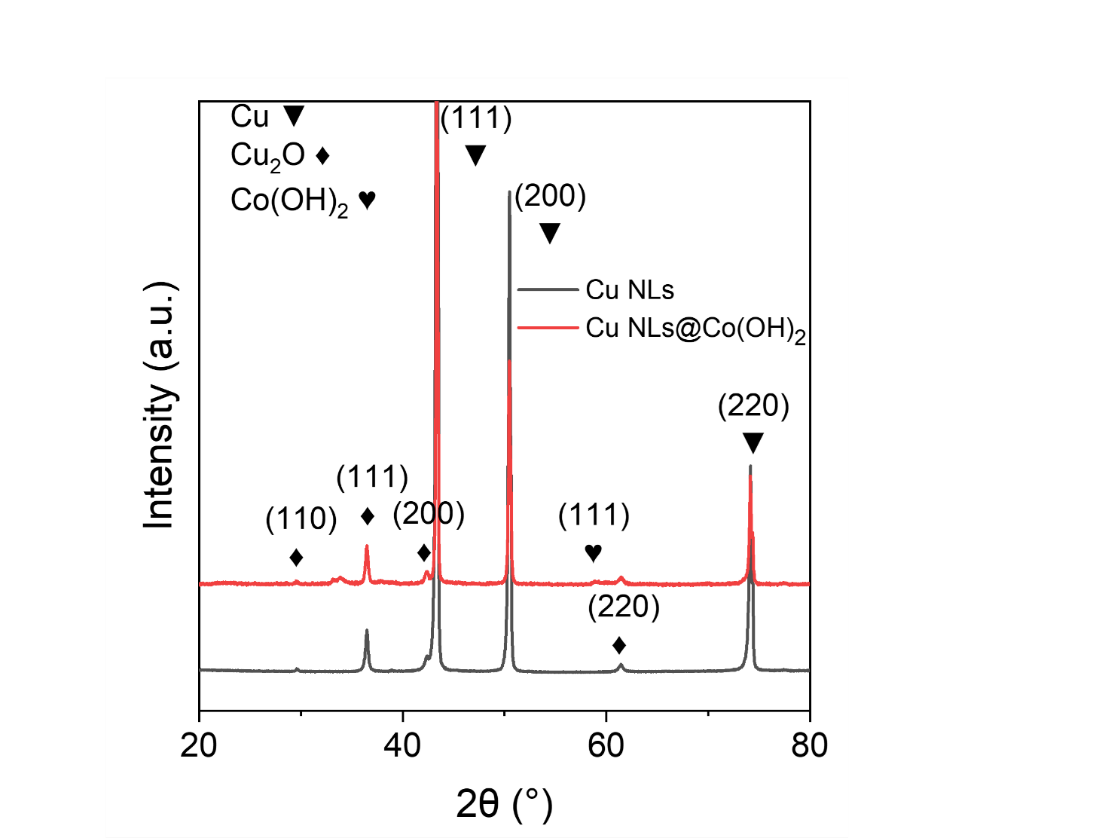


**Figure S4. X-ray diffraction spectroscopy of Cu NLs and Cu NLs@Co(OH)_2_.**


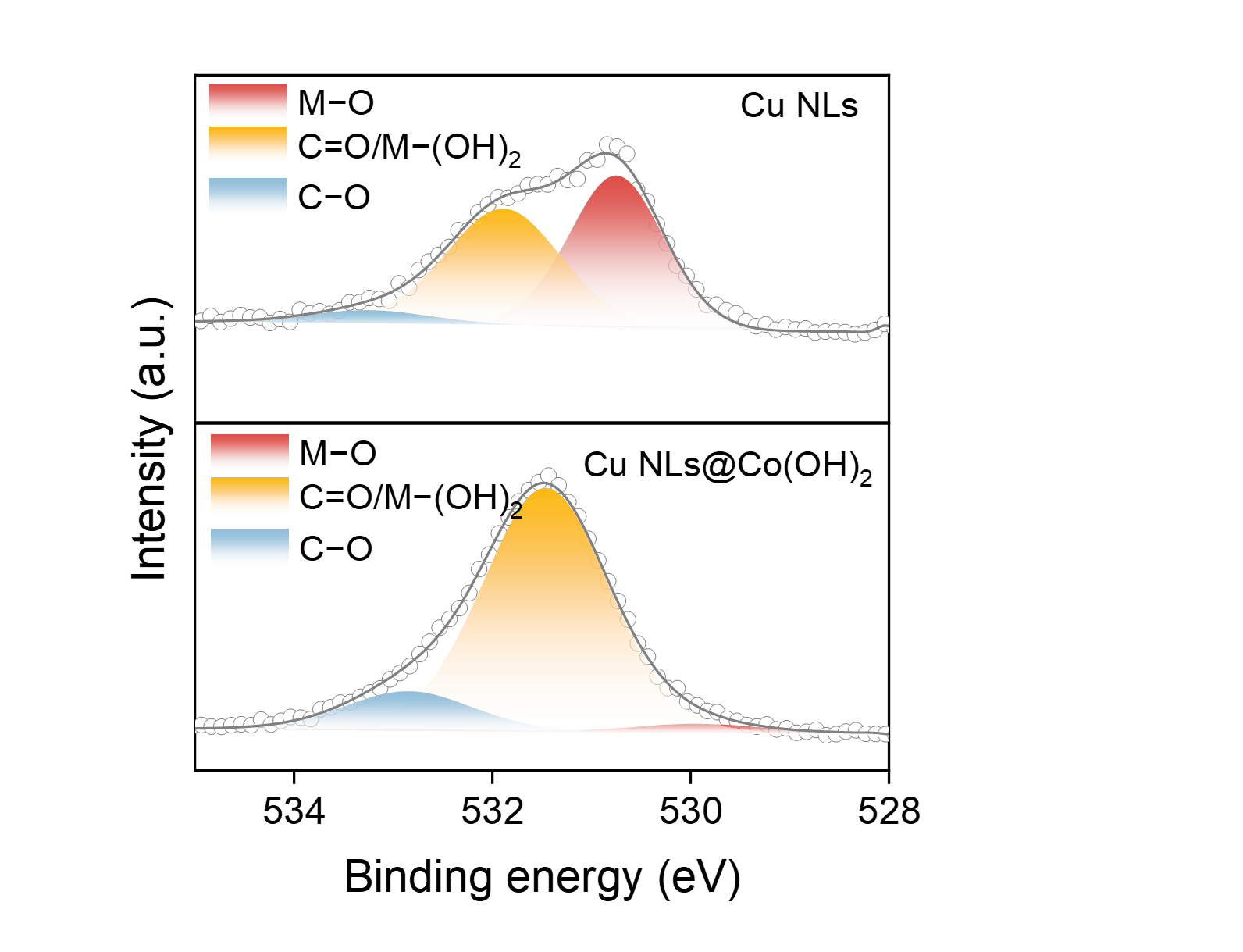


**Figure S5. High-resolution O 1*s* XPS spectra of Cu NLs and Cu NLs@Co(OH)_2_.**


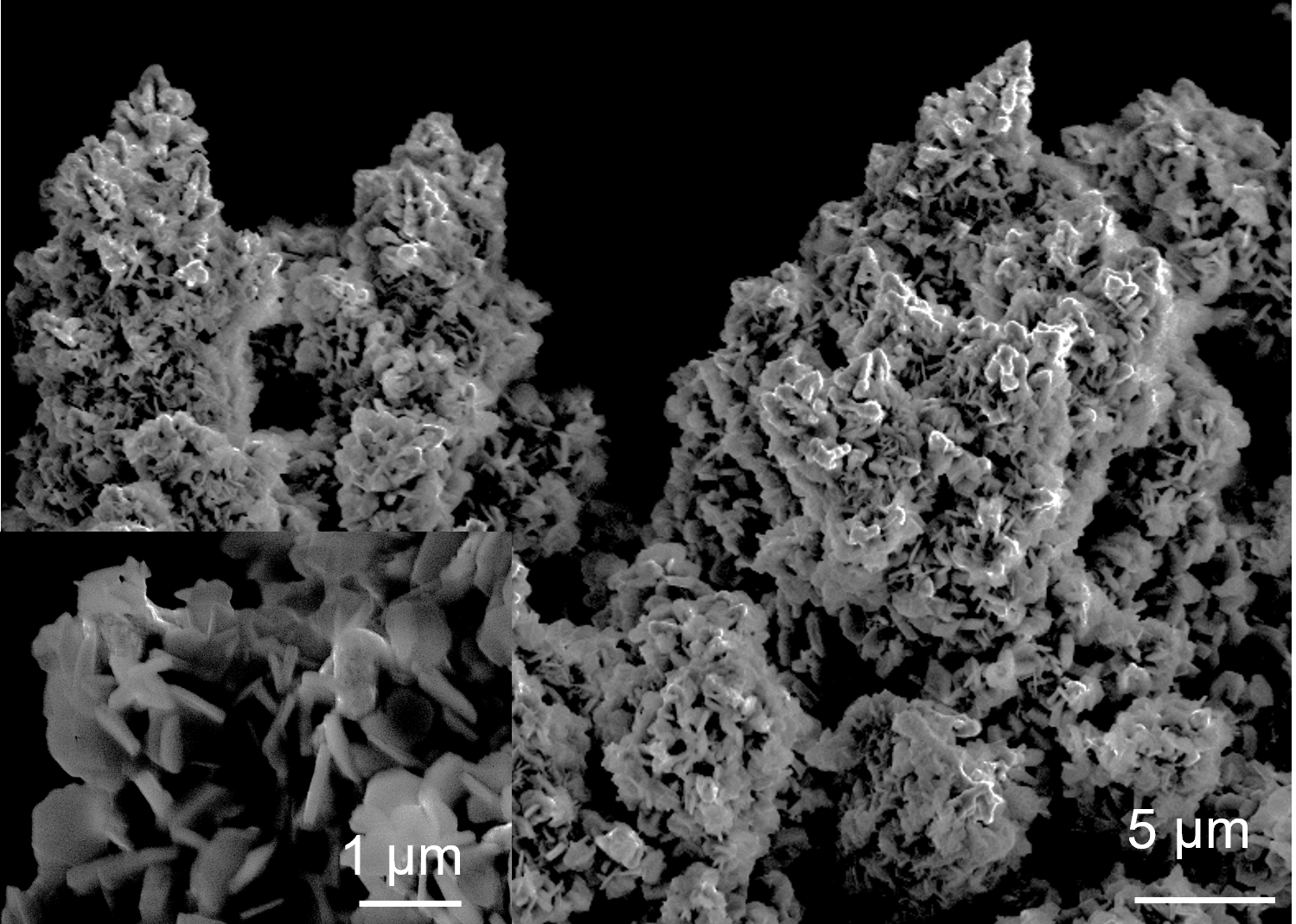


**Figure S6. SEM images of Cu NLs@Co(OH)_2_ post stability test.**


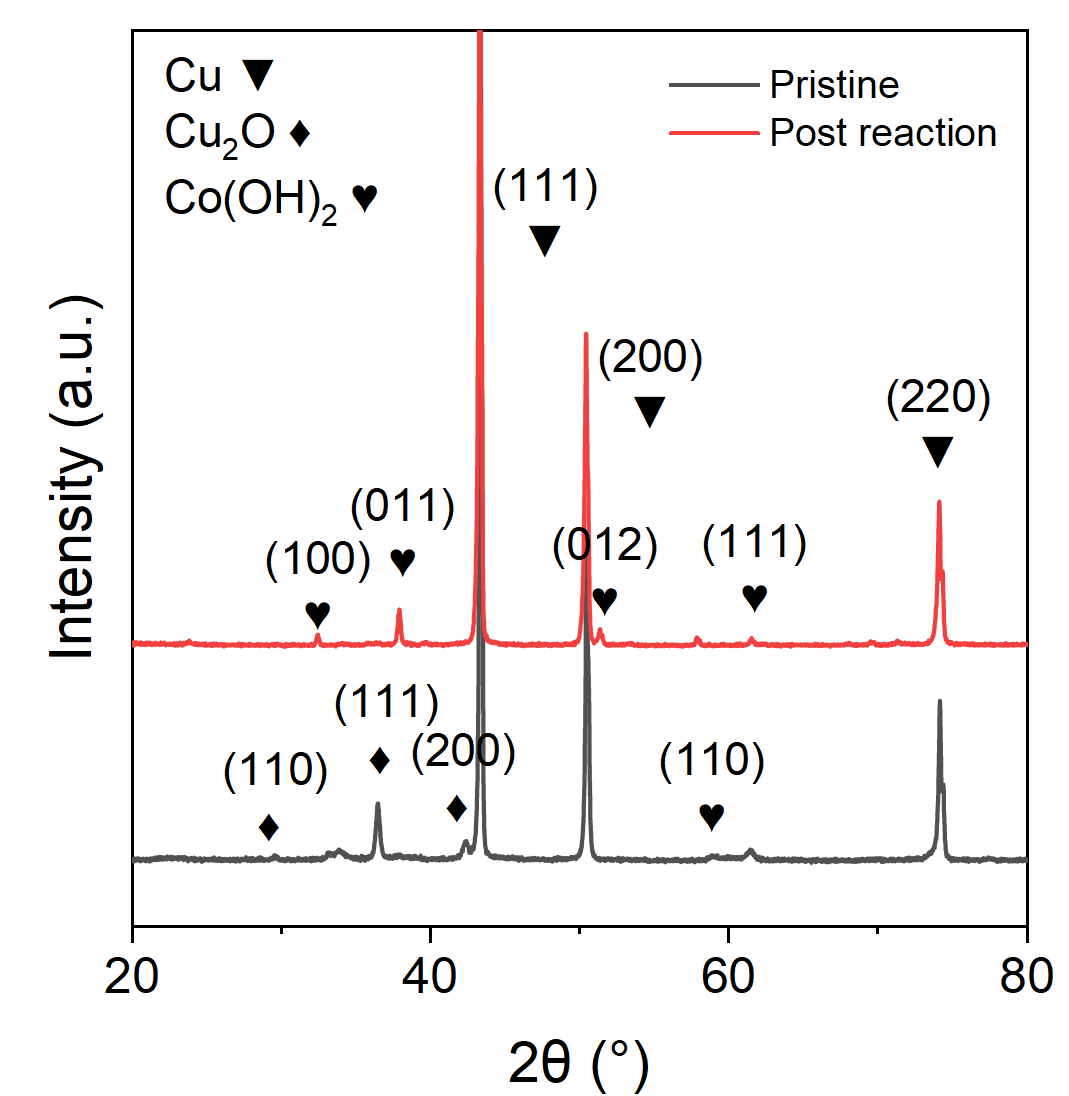


**Figure S7. X-ray diffraction spectroscopy of Cu NLs@Co(OH)_2_ post stability test.**


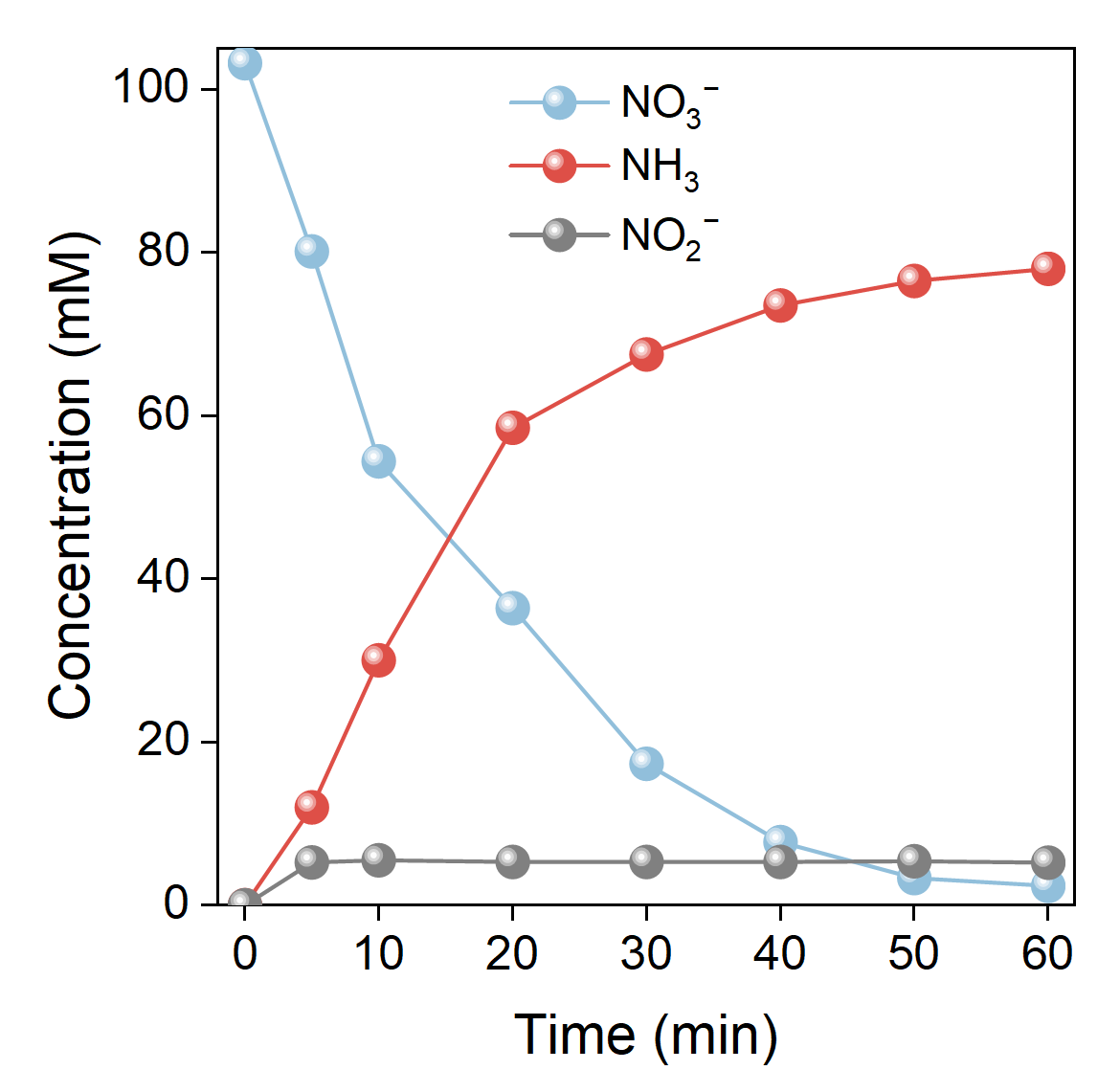


**Figure S8. The time-dependent concentration of NO_3_^−^, NO_2_^−^, and NH_3_ at −1.0 V vs RHE in 1 M KOH + 0.1 M KNO_3_. for Cu NLs@Co(OH)_2_.**


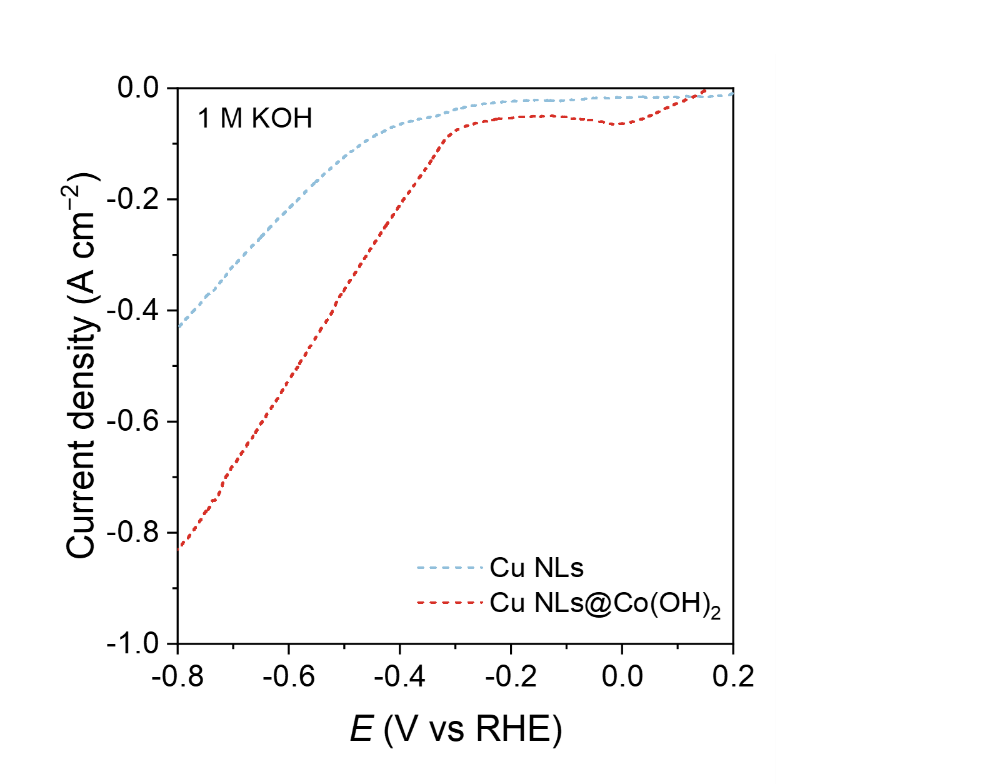


**Figure S9. HER performance of Cu NLs and Cu NLs@Co(OH)_2_.**


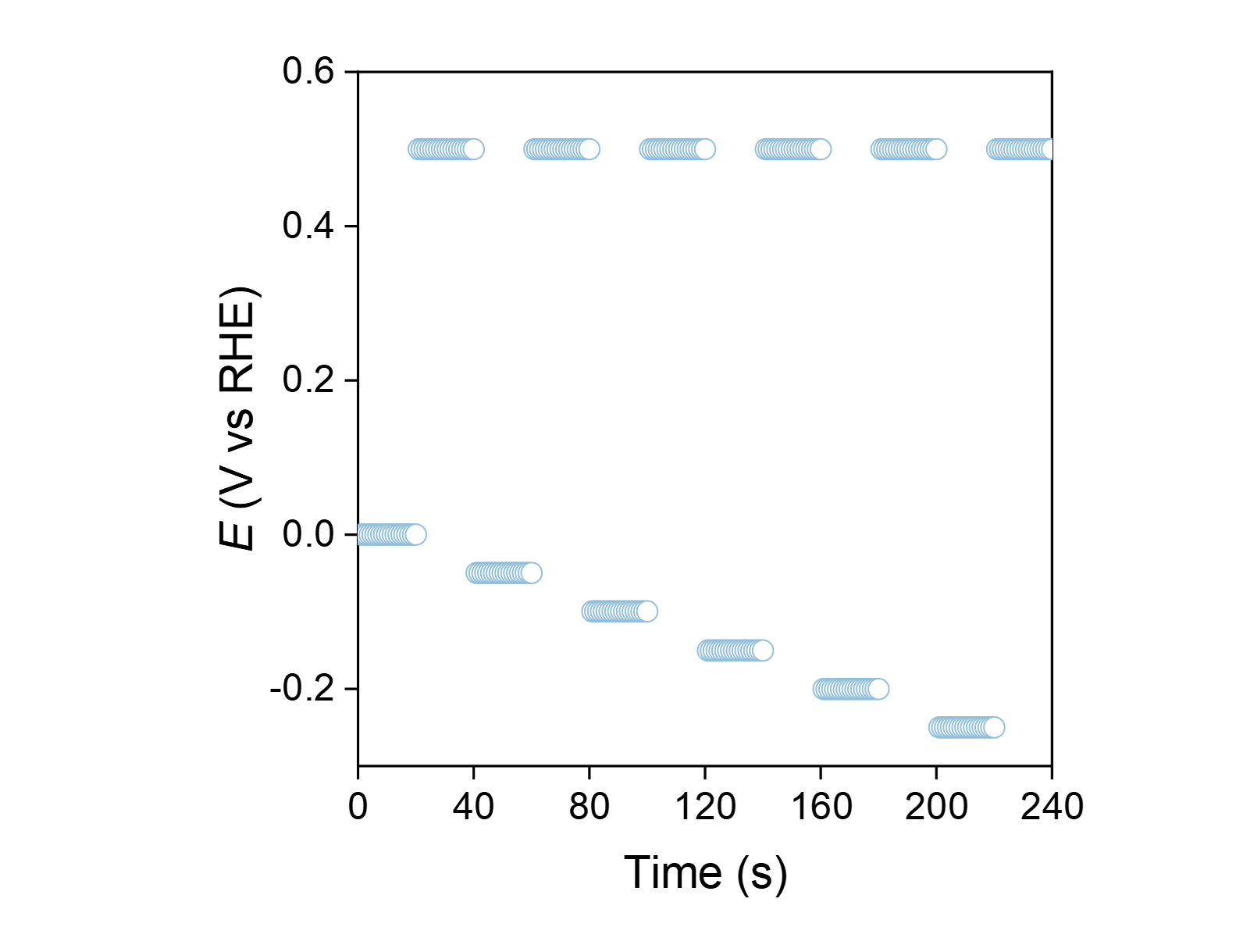


**Figure S10. The potential change curves of pulse voltammetry.**


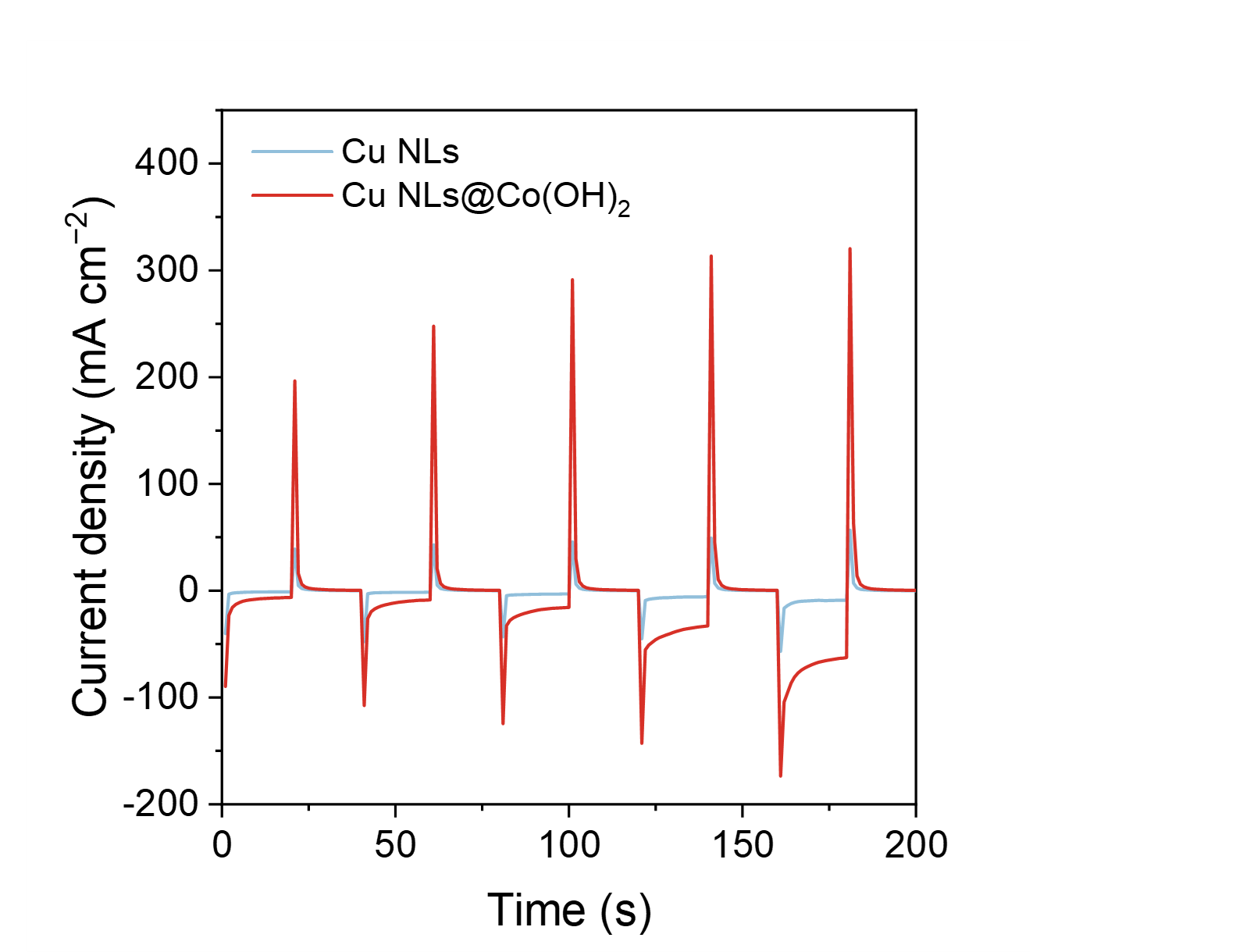


**Figure S11. The current change curve of pulse voltammetry.**


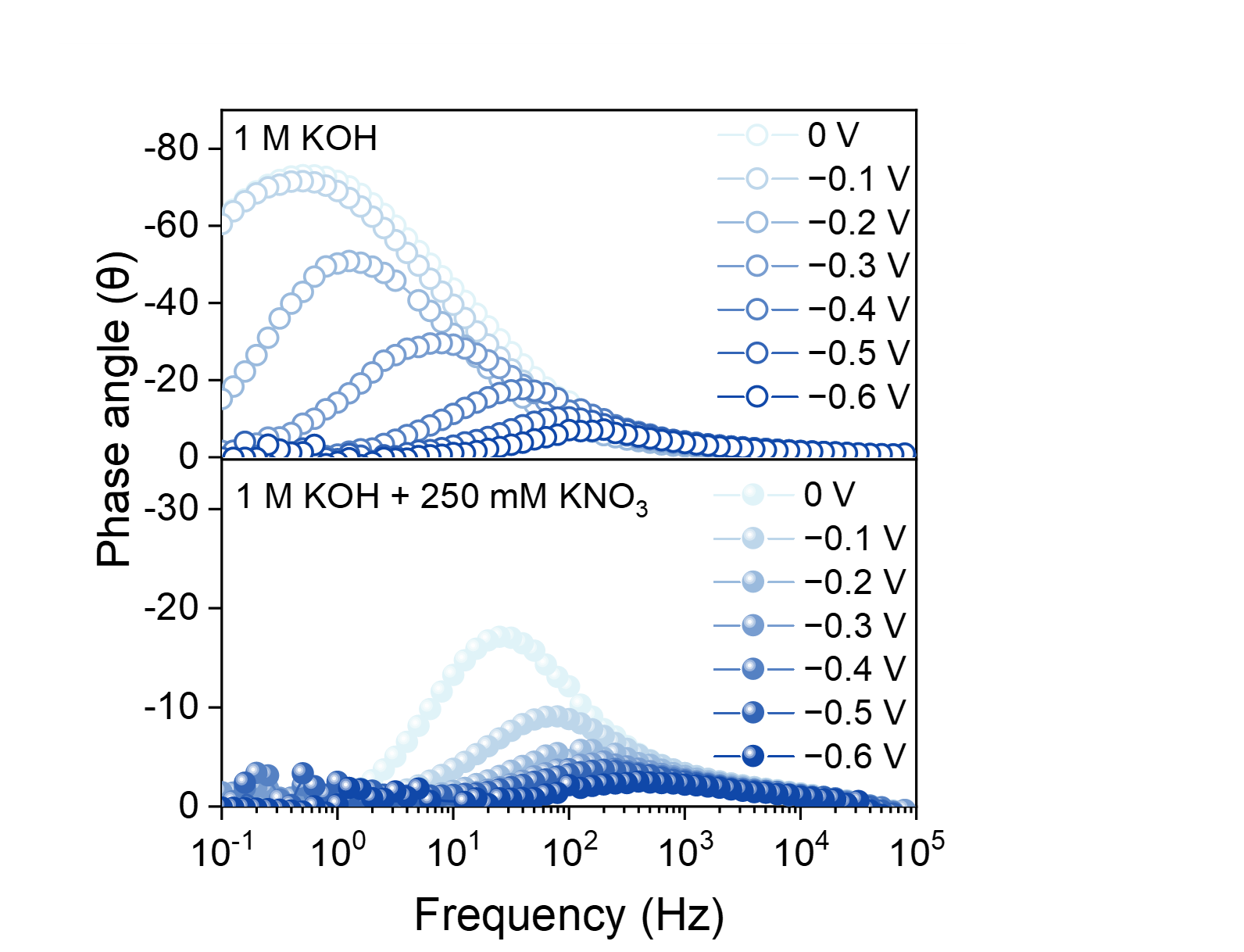


**Figure S12. Bode phase plots of Cu NLs at varied potentials in 1 M KOH with and without 250 mM NO_3_^−^.**

**
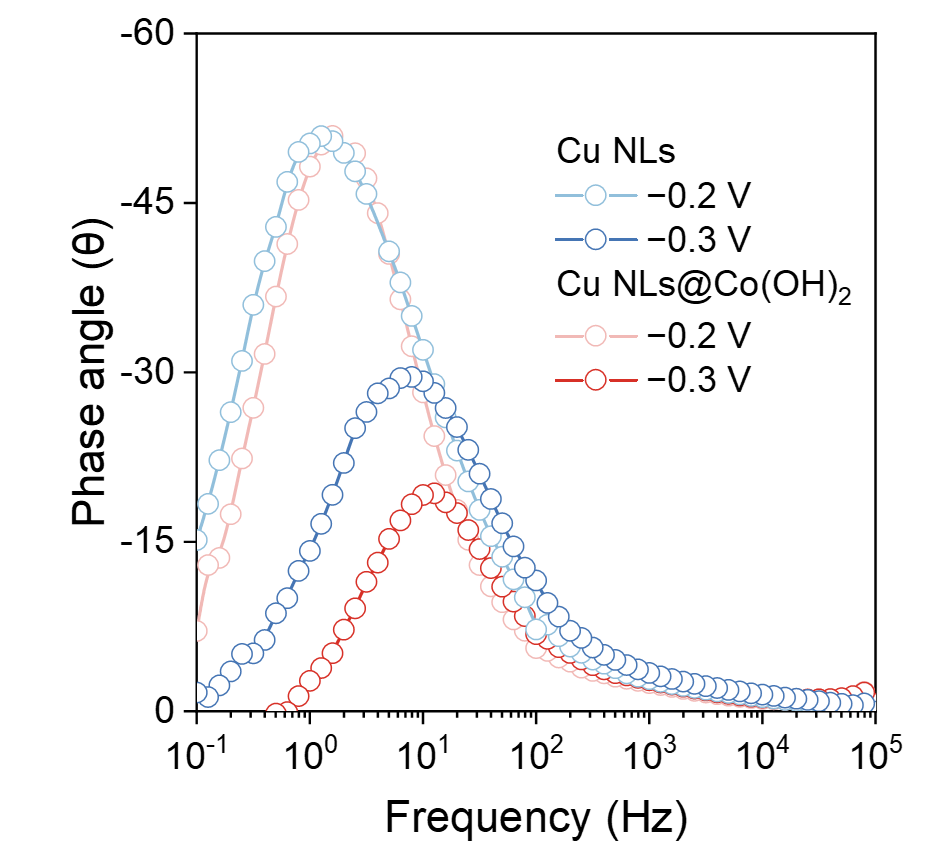
**

**Figure S13. Bode phase plots of Cu NLs and Cu NLs@Co(OH)_2_ in 1 M KOH.**


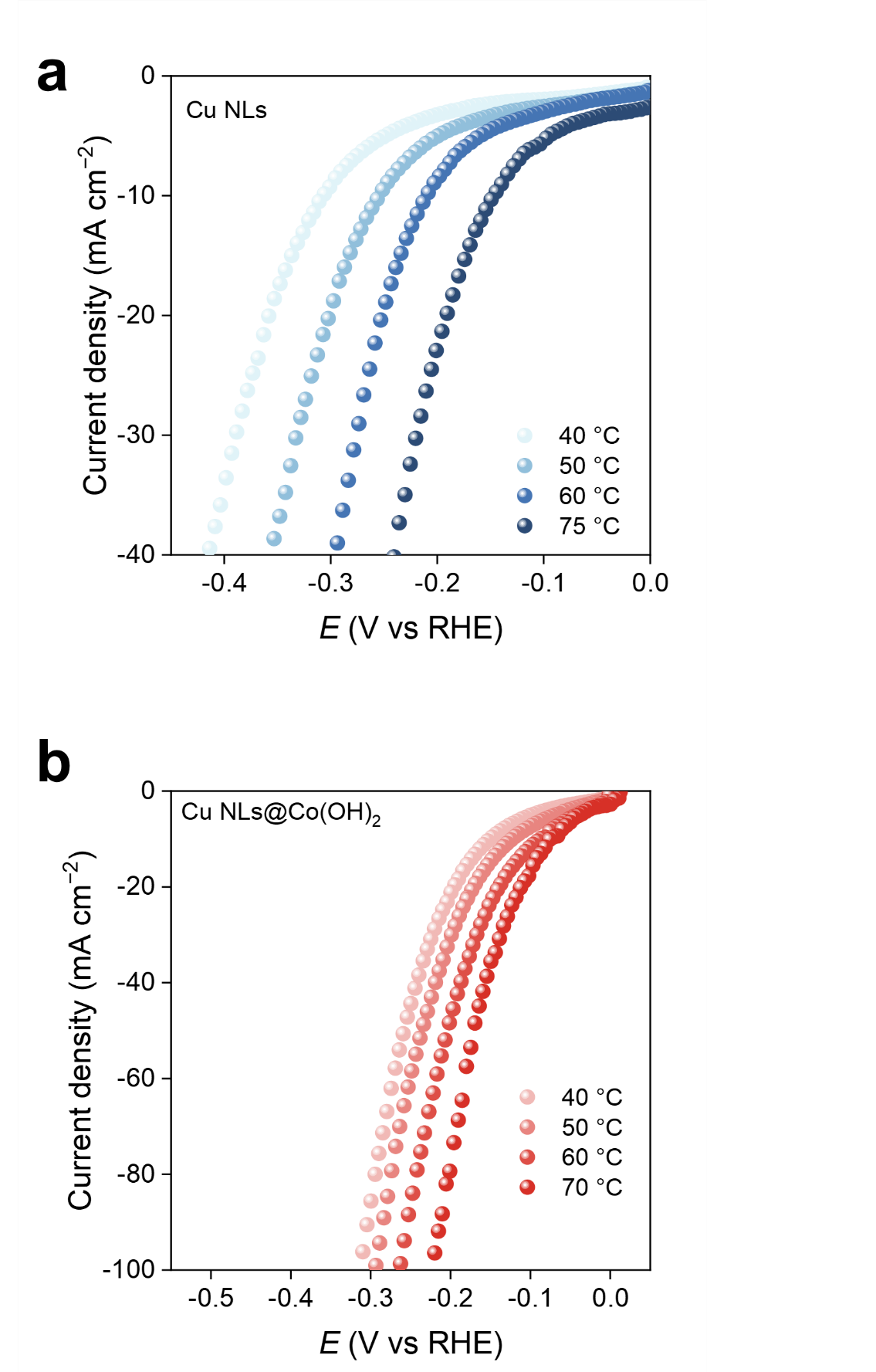


**Figure S14. Temperature dependent HER performance of Cu NLs and Cu NLs@Co(OH)_2_.**

**a**) linear sweep voltammetry **(**LSV) curves of Cu NLs in 1 M KOH. **b**) LSV curves of Cu NLs@Co(OH)_2_.


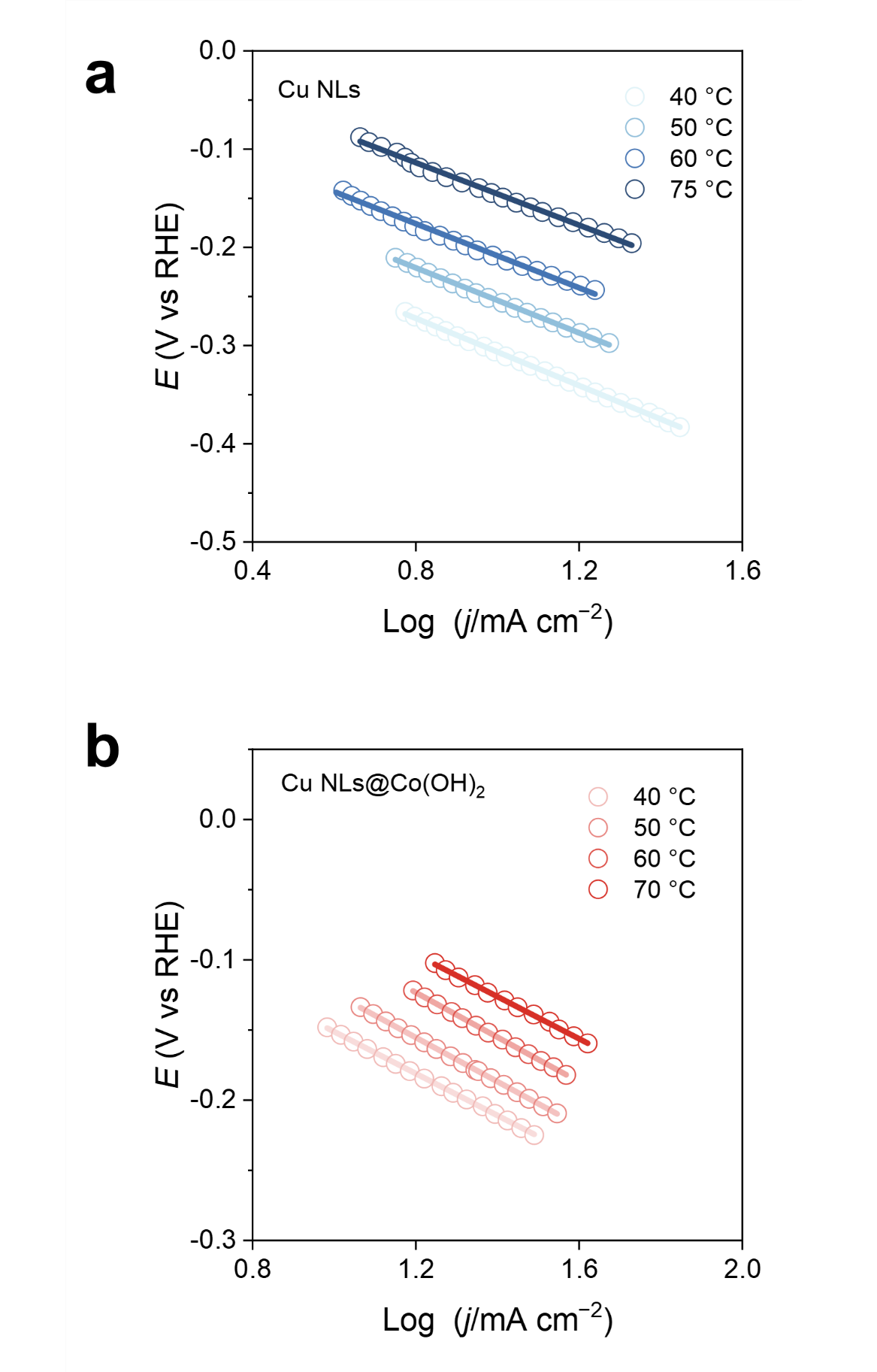


**Figure S15. Tafel plots of Cu NLs and Cu NLs@Co(OH)_2_.**

**a**) Cu NLs **b**) Cu NLs@Co(OH)_2_.


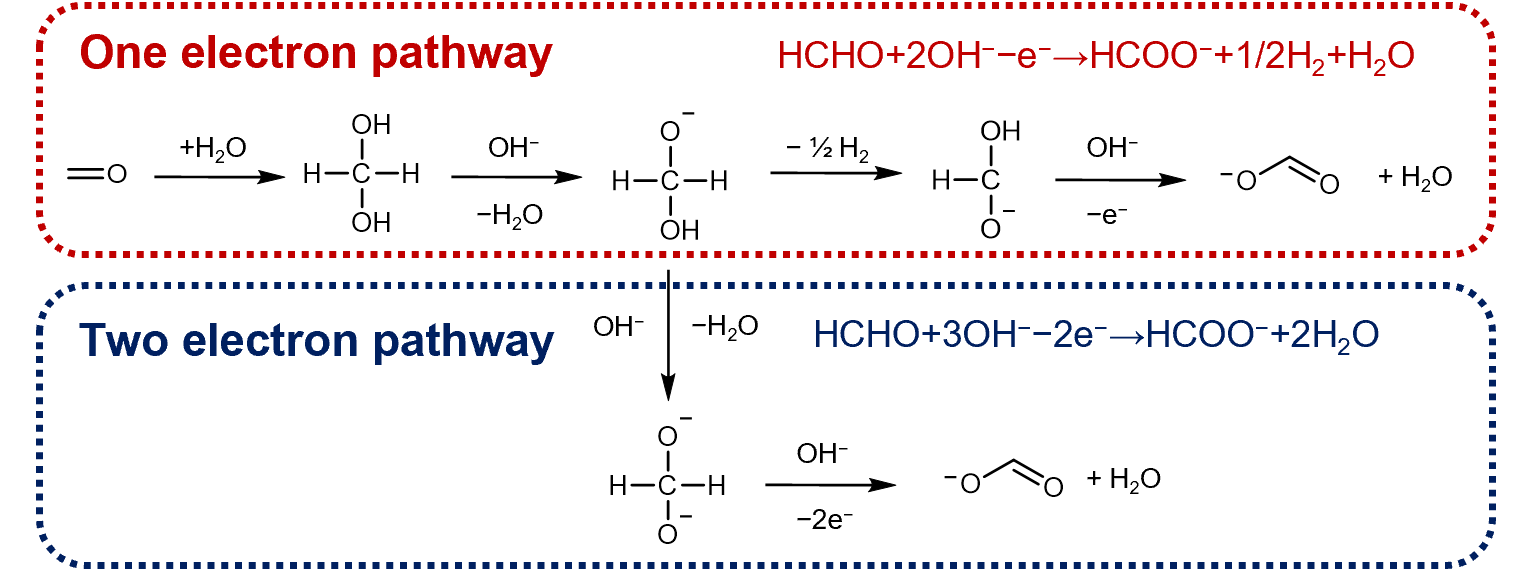


**Figure S16. Schematic diagrams for the formaldehyde oxidation pathways.**


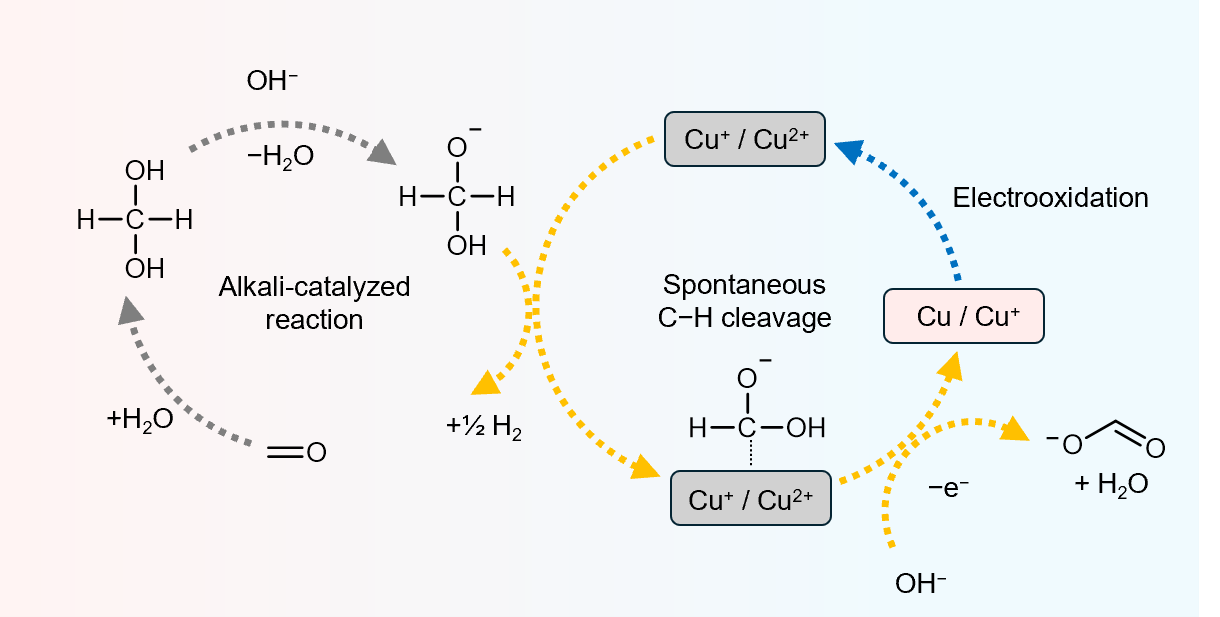


**Figure S17. Formaldehyde oxidation reaction mechanism over Cu NLs@Co(OH)_2_.**


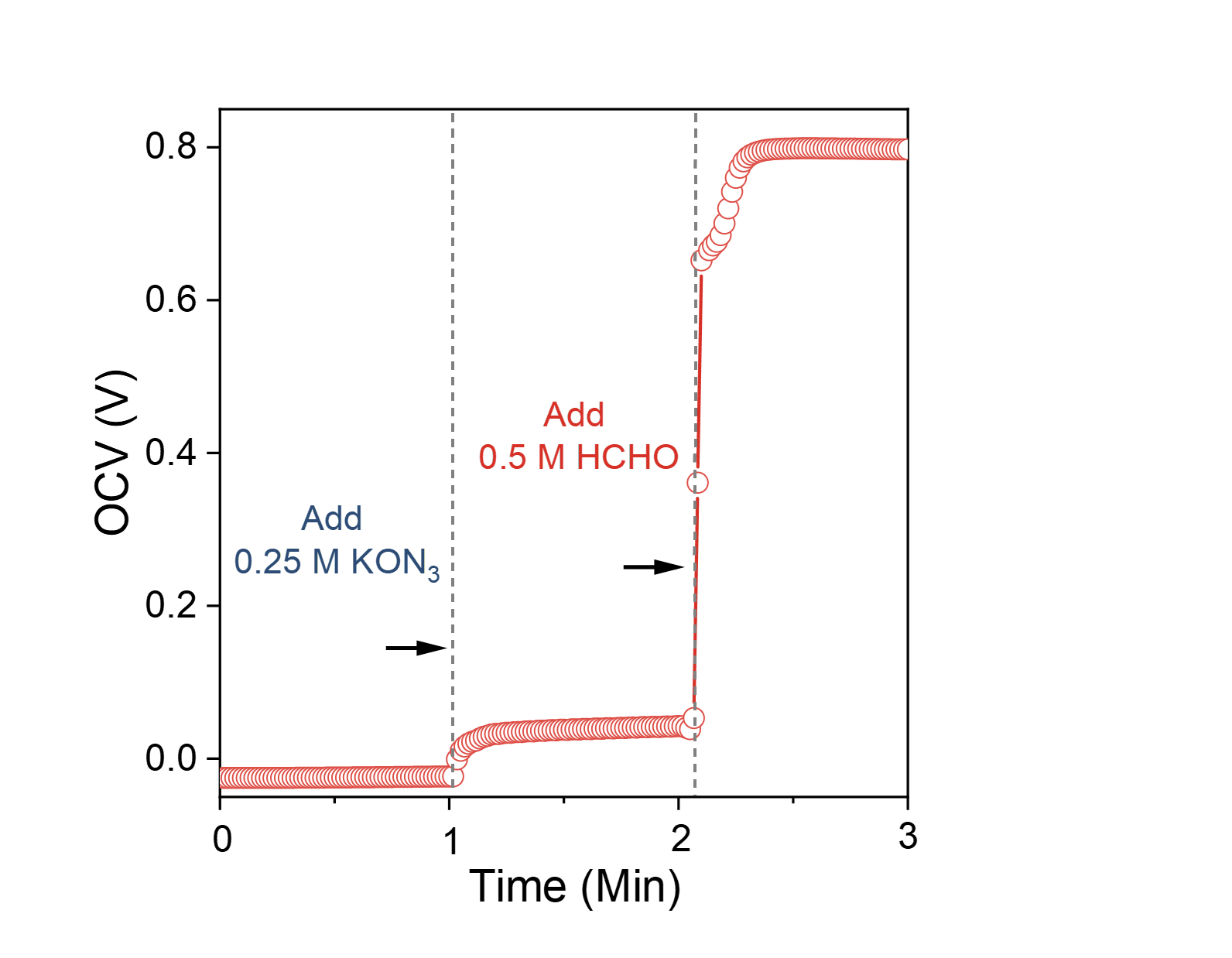


**Figure S18. OCV changes curve of AFCFC.**


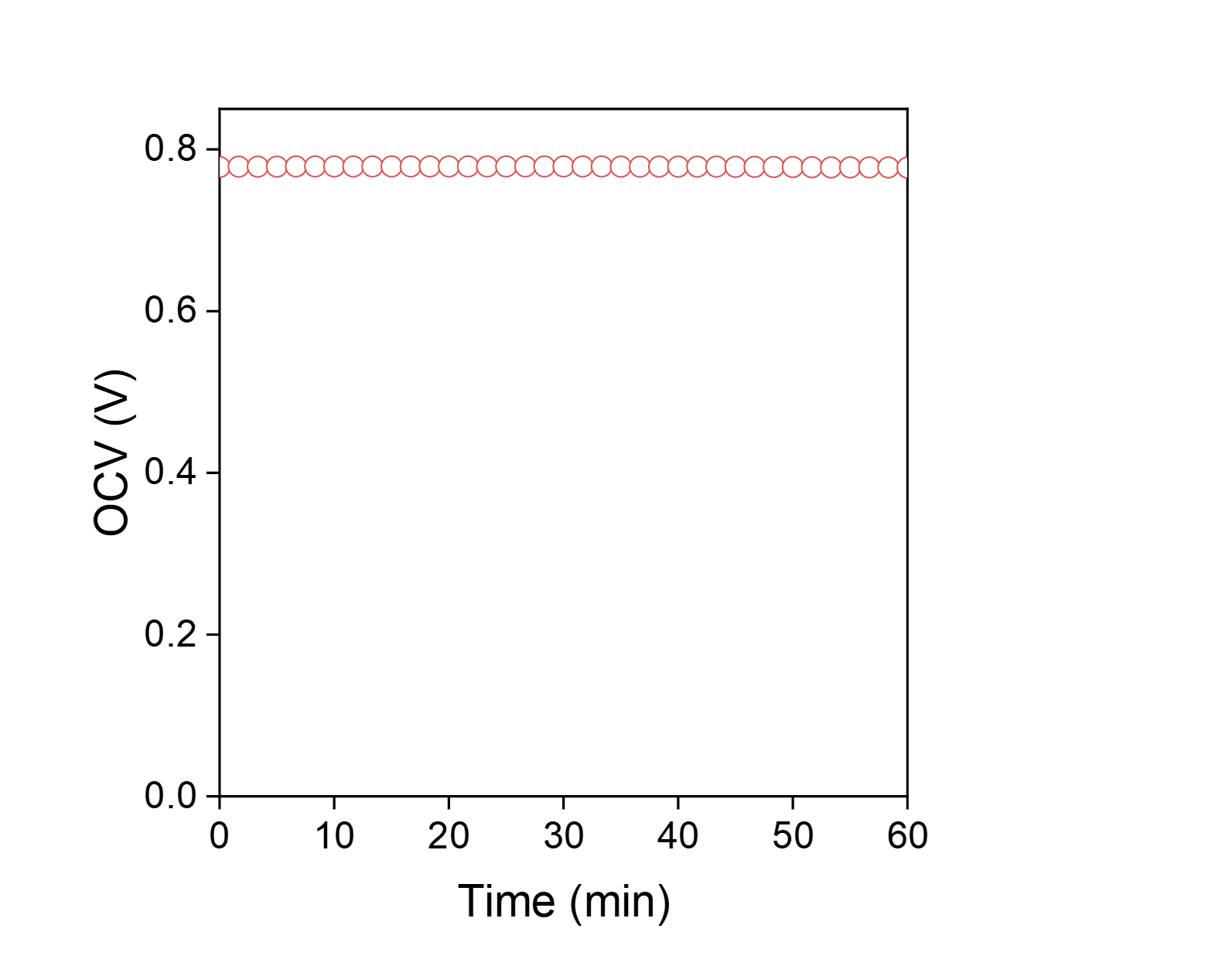


**Figure S19. OCV stability curve of AFCFC.**


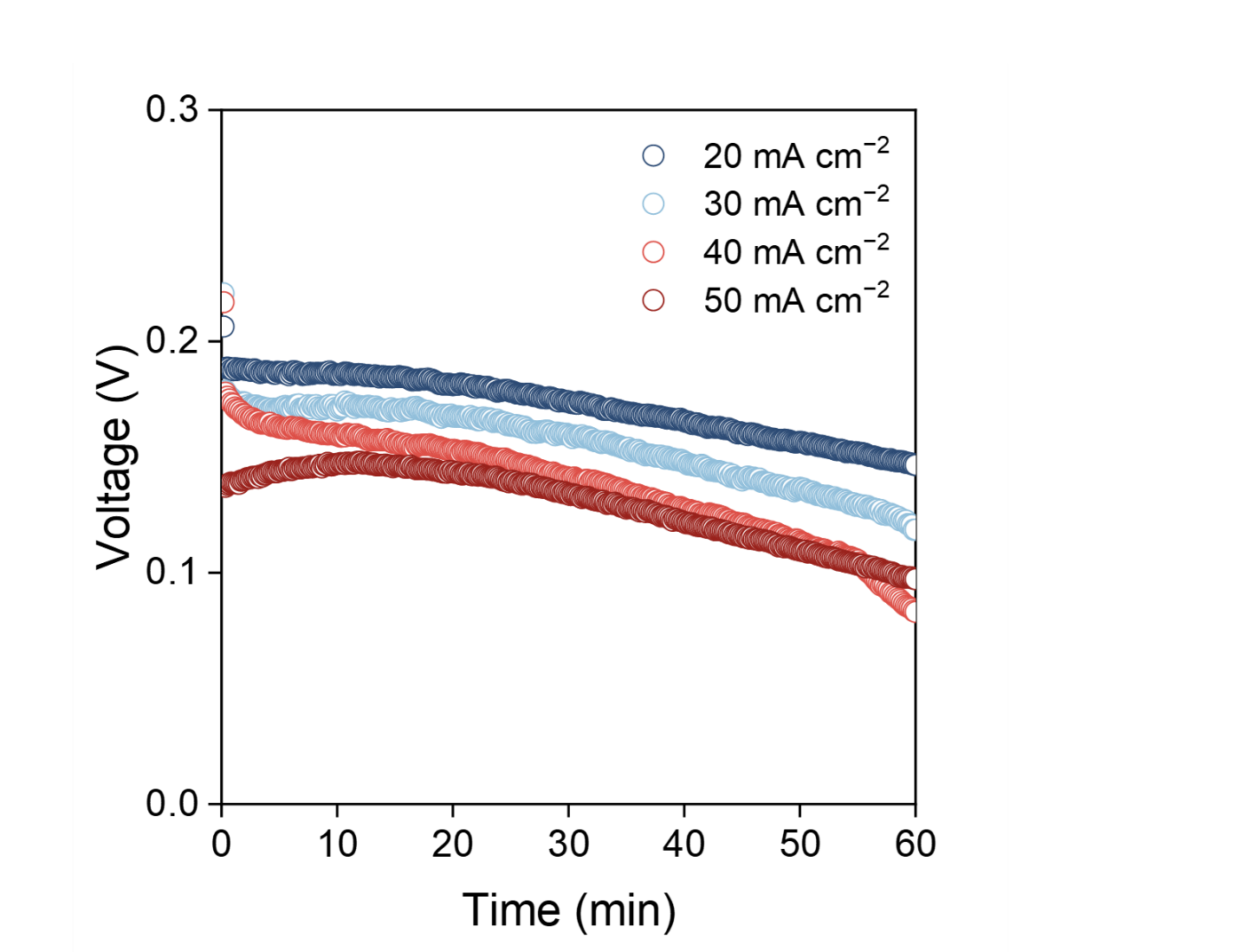


**Figure S20. Discharge curve of AFCFC with different current density.**


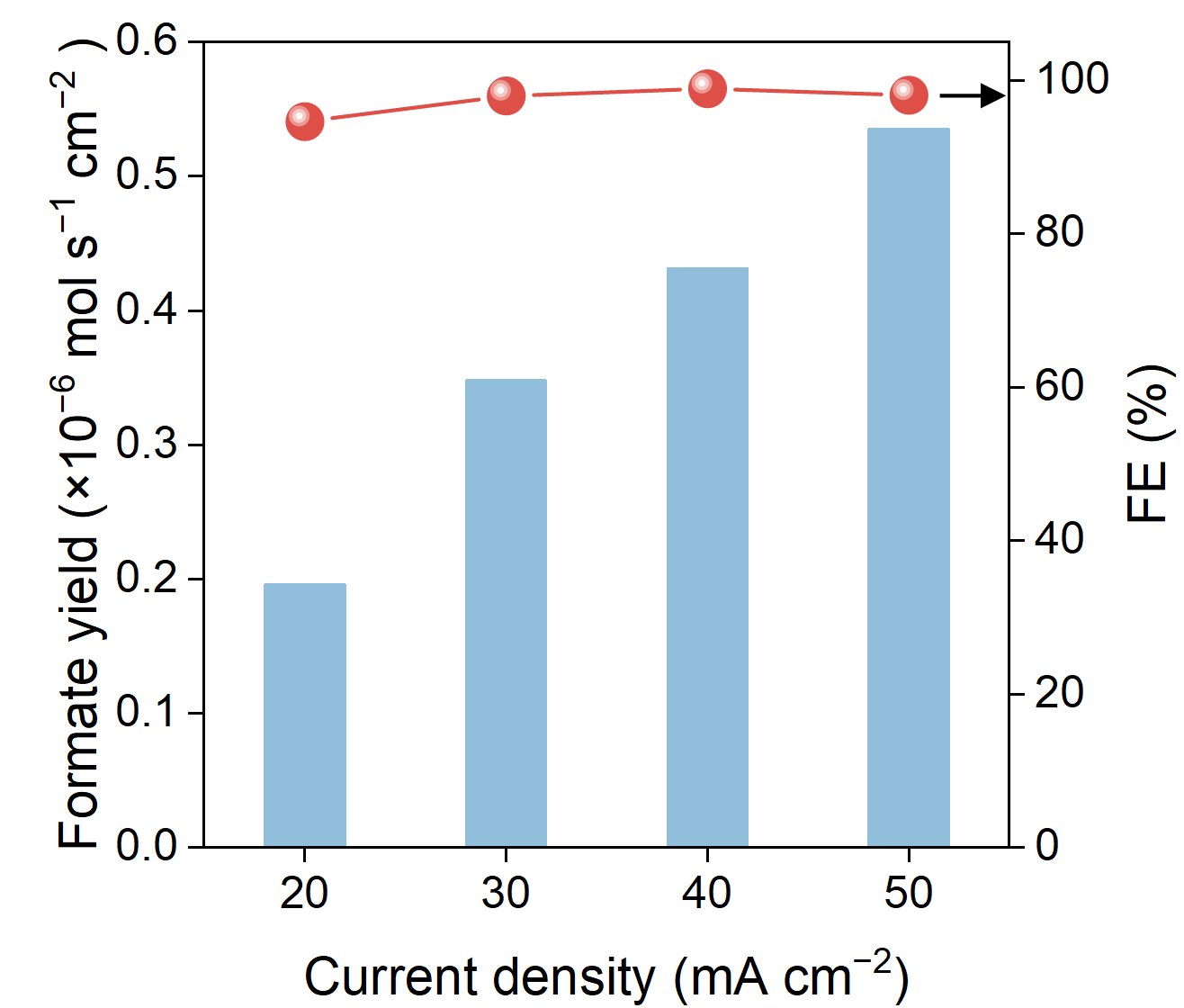


**Figure S21. FEs towards formate and formate yield rates of AFCFC.**


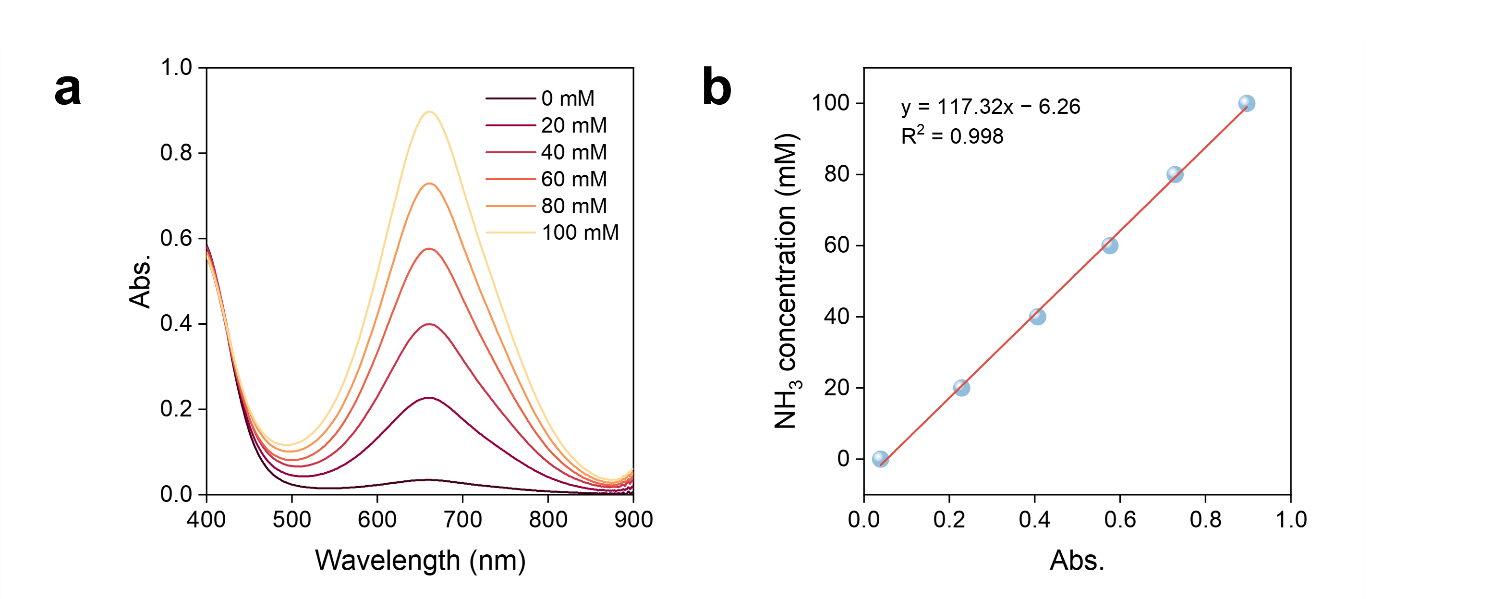


**Figure S22. NH_3_ quantification using UV-vis absorption spectroscopy.**

**a**) UV-vis absorption spectra. **b**) Standard calibration curve for NH_3_.


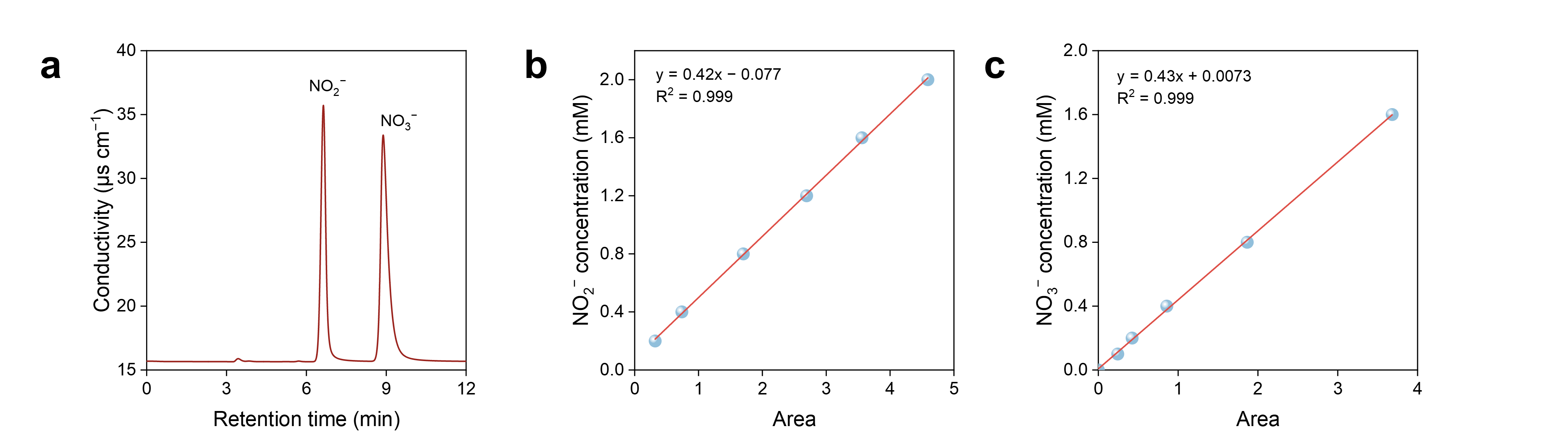


**Figure S23. NO_2_^−^ and NO_3_^−^ quantification using ion chromatography.**

**a**) ion chromatography spectra. **b**) Standard calibration curve for NO_2_^−^. **c**) Standard calibration curve for NO_3_^−^.


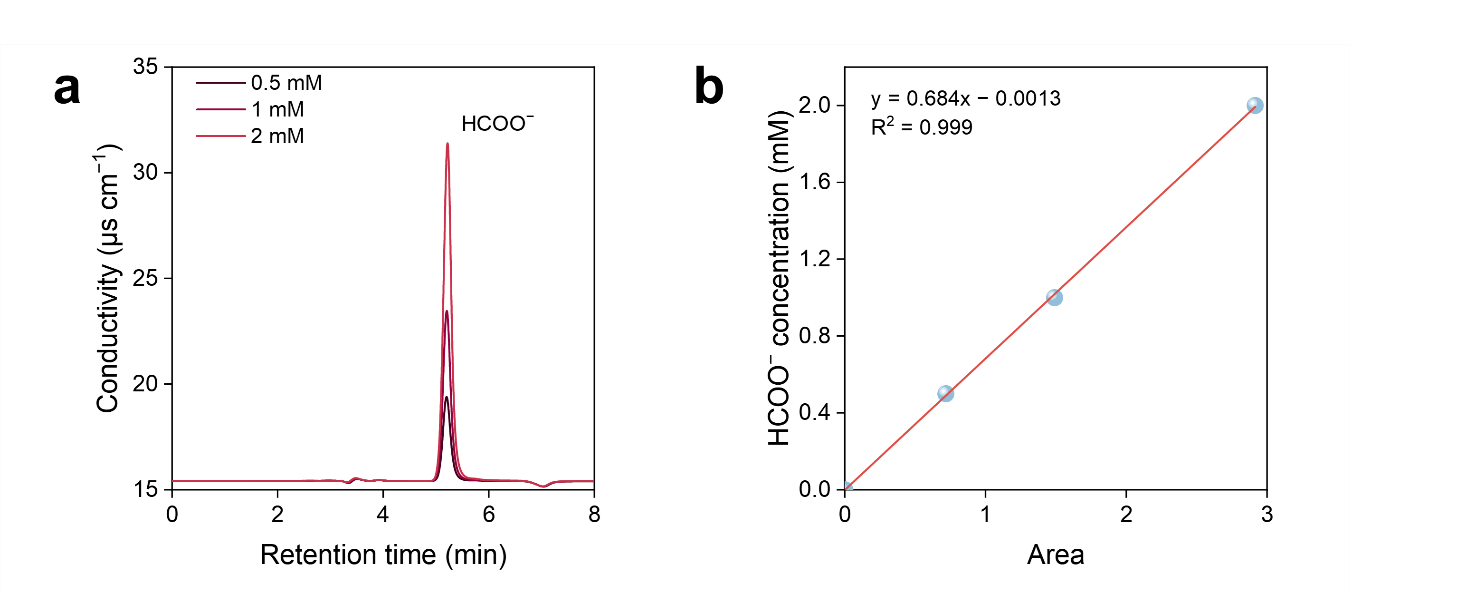


**Figure S24. formate quantification using ion chromatography.**

**a**) ion chromatography spectra. **b**) Standard calibration curve for formate.


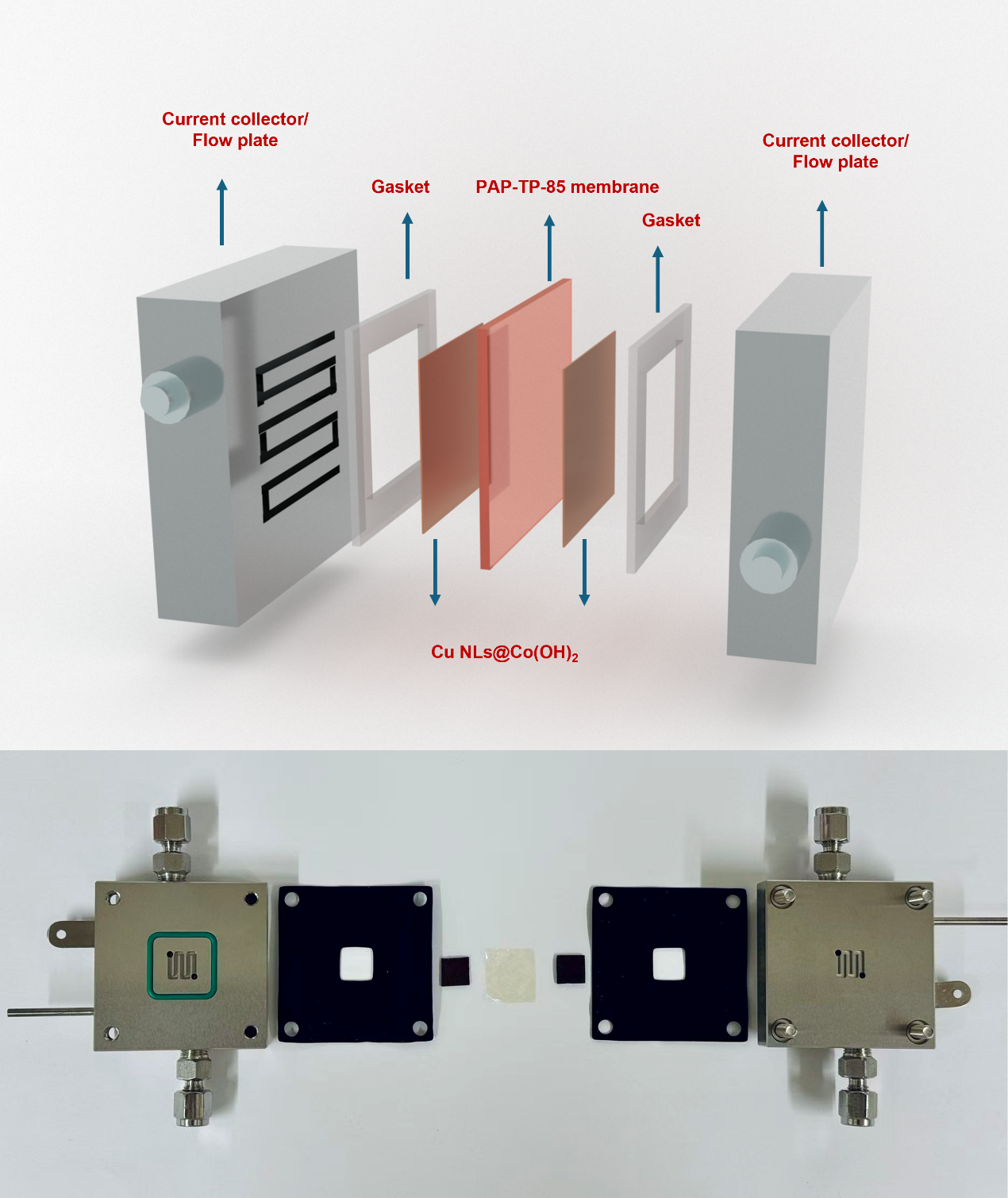


**Figure S25. Schematic diagram and digital photo for the AFCFC device.**


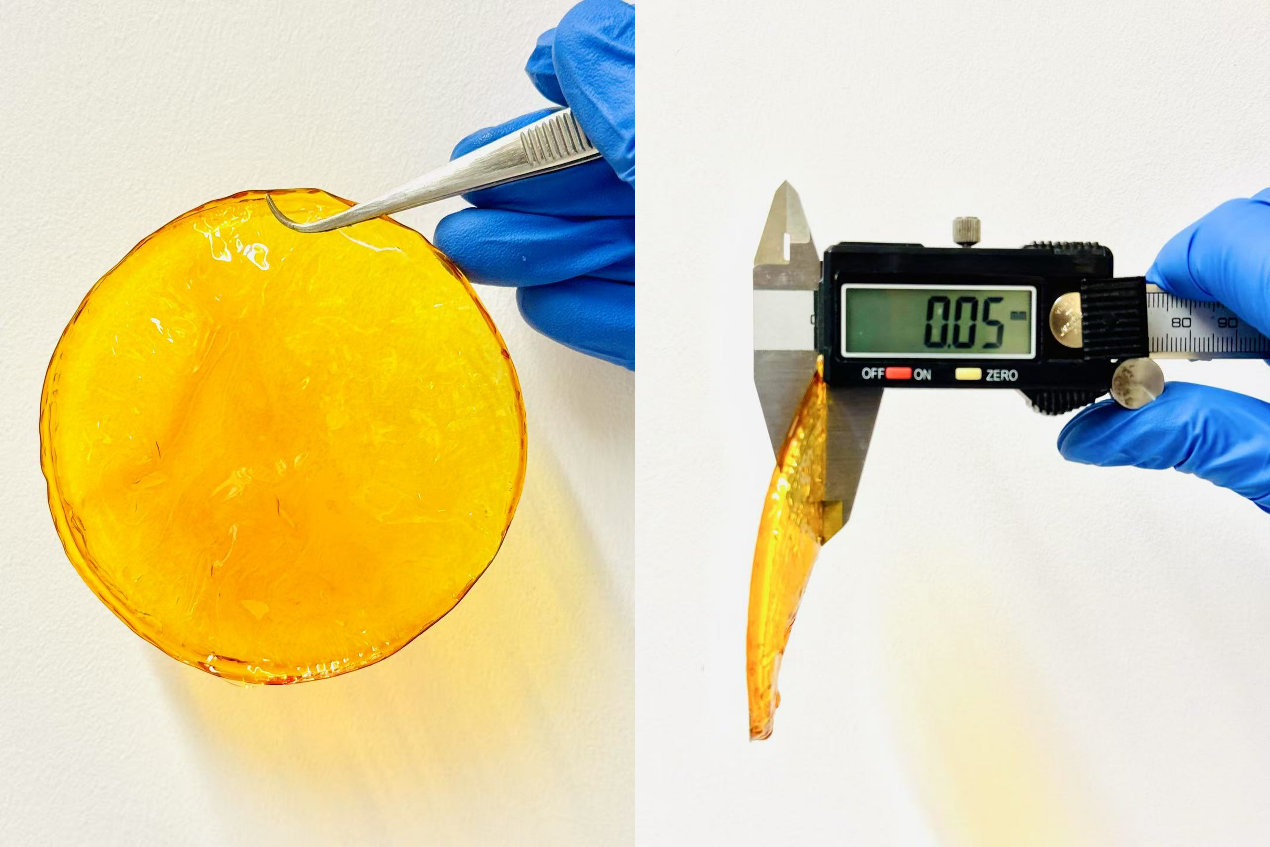


**Figure S26. Digital photos of home-made PAP-TP-85-N membrane.**


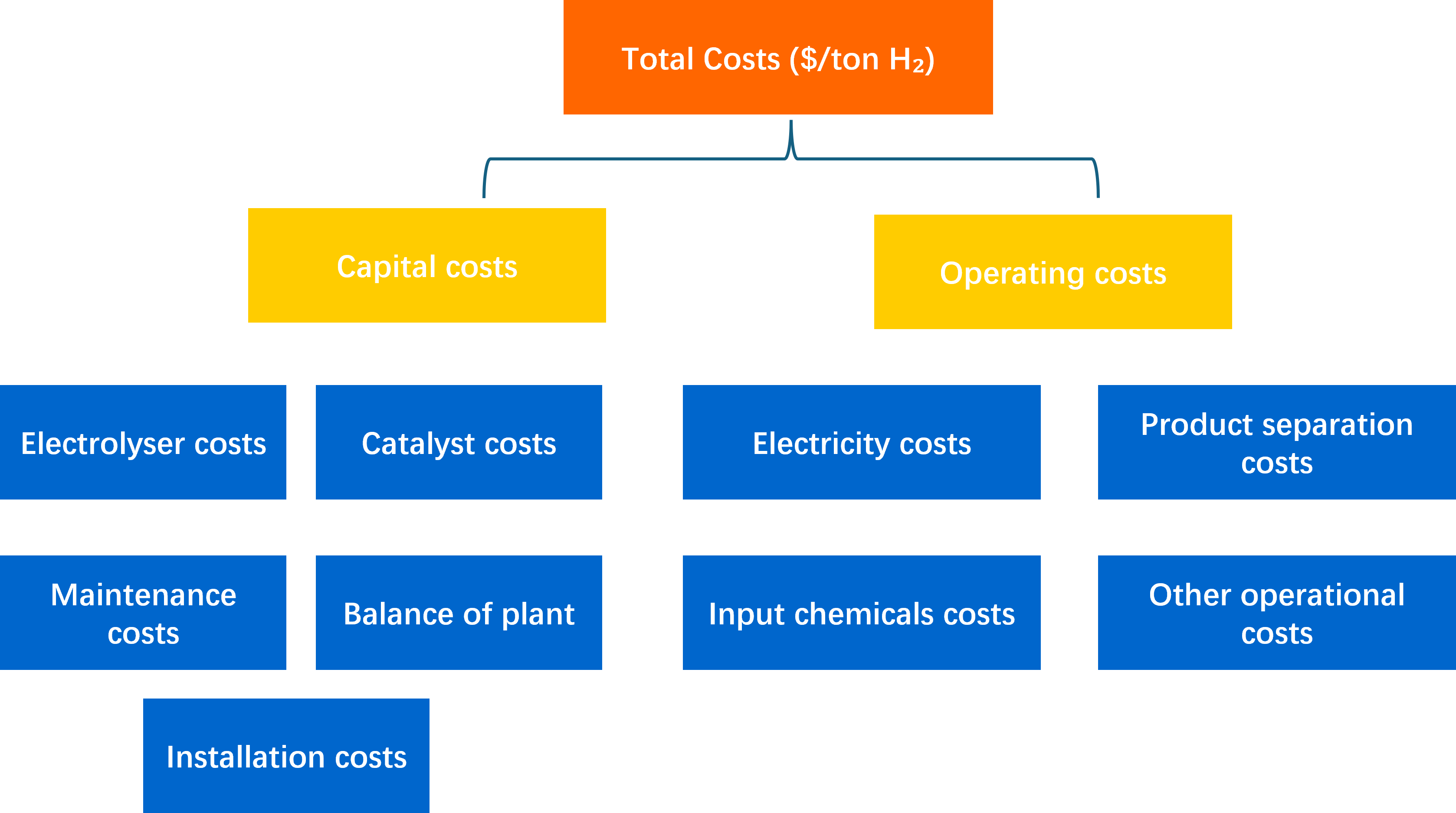


**Figure S27. Model of the techno-economic analysis of electrochemical ammonia production.**


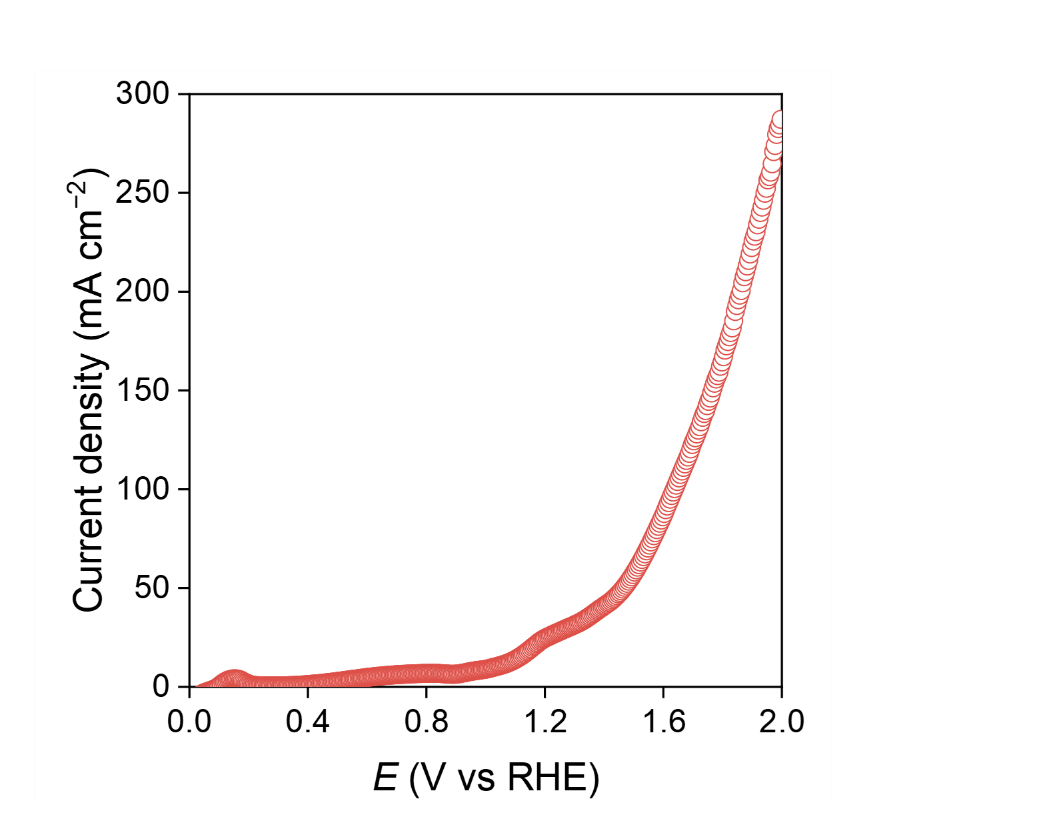


**Figure S28. The polarisation curve of NO_3_RR coupled with OER.**

**Supplementary Tables**

**Table S1. XPS composition reports of Cu NLs and Cu NLs@Co(OH)_2_.**

| Sample | Cu | O | C | Co |
| --- | --- | --- | --- | --- |
| Cu | 20.90 | 37.38 | 41.71 | - |
| Cu@Co(OH)_2_ | 1.01 | 50.80 | 25.64 | 22.57 |

**Table S2. Comparison of the NO_3_RR performance of Cu NLs@Co(OH)_2_ with other reported electrocatalysts in recent years.**

| Electrocatalyst | NO_3_^−^ Conc. | Potential (V) | FE (%) | *j*max. (mA cm^−2^) | NH_3_ yield rate (×10^−6^ mol s^−1^ cm^−2^) | Journal/year | Ref. |
| --- | --- | --- | --- | --- | --- | --- | --- |
| Ag_1_@Cu_2_O NWs | 0.5 M | −1.0 | 91.2% | 2320 | 3.01307 | *Energy Environ. Sci./*2025 | ^[3]^ |
| CuCo_2_O_4_/Ni | 0.2 M | −0.9 | 96.8% | 1384.7 | 2.38235 | *Adv. Mater.*/2023 | ^[4]^ |
| CNS-CoP/Cu foam | 1.0 M | −1.03 | 88.6% | ~550 | 2.35294 | *Nat. Commun.*/2022 | ^[5]^ |
| Ru/Cu_2_O | 1.0 M | −0.4 | 75% | 2007.5 | 1.94444 | *J. Am. Chem. Soc.*/2023 | ^[6]^ |
| Co_3_O_4_/CuI-N-C | 1.0 M | −1.0 | 97.7% | ~1700 | 1.86275 | *Nat. Commun.*/2024 | ^[7]^ |
| Au*_x_*Cu SAAs | 0.1 M | −0.8 | ~100% | ~1500 | 1.725 | *Angew. Chem. Int. Ed.*/2025 | ^[8]^ |
| CuNi NPs/CF | 0.715 M | −0.68 | 97.03% | ~1350 | 1.54575 | *Energy Environ. Sci.*/2023 | ^[9]^ |
| 0.6W-O-CoP@NF | 0.1 M | −0.7 | 95.2% | ~950 | 1.45261 | *Adv. Mater.*/2023 | ^[10]^ |
| Cu_5_Co_5_O | 0.1 M | −1.0 | ~100% | ~1000 | 1.33333 | *Nat. Commun.*/2022 | ^[11]^ |
| Ru-Cu NW | 0.032 M | −0.2 | 93% | ~1000 | 1.25 | *Nat. Nanotech.*2022 | ^[12]^ |
| Cu-N_4_B_2_ | 0.1 M | −0.7 | 98.2% | ~300 | 1.25 | *Energy Environ. Sci.2024* | ^[13]^ |
| Cu plates | 0.5 M | −2.0 | 99% | 900 | 0.87255 | *Angew. Chem. Int. Ed.*/2024 | ^[14]^ |
| (Co_0.83_N_i0.16_)_2_Fe | 0.1 M | −0.6 | 97.8% | ~700 | 0.82516 | *Angew. Chem. Int. Ed.*/2024 | ^[15]^ |
| D-Ni(OH)_2_/Ni@CF | 0.1 M | −0.7 | 98.99% | ~560 | 0.78186 | *Nat. Commun.*/2024 | ^[16]^ |
| CNS@CoP | 0.1 M | −1.0 | 95.1% | ~450 | 0.71732 | *Chem Catal.*/2023 | ^[17]^ |
| P-Cu/Co(OH)_2_ | 0.1 M | −0.4 | 97.04% | ~2000 | 0.69657 | *Adv. Mater.*/2024 | ^[18]^ |
| FeB_2_ | 0.1 M | −0.6 | 96.8% | ~322 | 0.41667 | *Angew. Chem. Int. Ed.*/2023 | ^[19]^ |
| CuPd nanocubes | 1.0 M | −0.7 | 92.5% | ~400 | 0.34804 | *Nat. Commun.*/2022 | ^[20]^ |
| Cu NLs@Co(OH)_2_ | 0.25 M | −1.0 | 92.32% | 2150 | 2.03 | - | This work |
|  | 0.5 M | −1.1 | ~100% | 2235 | 2.67 |  |  |
|  | 1 M | −1.2 | 96.82% | 2350 | 3.18 |  |  |

**Table S3. Comparison of the performance of AFCFC with other reported unassisted NH_3_ synthesis system in recent years.**

| Anode//Cathode | OCV (V) | Current density (mA cm^−2^) | Power density (mW cm^−2^) | Stability@ Current density (hours@mA cm^−2^) | Journal/year | Ref. |
| --- | --- | --- | --- | --- | --- | --- |
| Ag_1_@Cu_2_O NWs//Ag_1_@Cu_2_O NWs | 0.52 | 80.3 | 3.6% | - | *Energy Environ. Sci./*2025 | ^[3]^ |
| CuAg-3:7//CuRu-9:1 | 0.75 | 29.5 | 3.38 | 50@10 | *Angew. Chem. Int. Ed.*/2024 | ^[21]^ |
| Pd-Cu_3_P SA-QDs//Pd-Cu_3_P SA-QDs | 0.892 | ~50 | 12.1 | 25@~15 | *Adv. Mater.*/2025 | ^[22]^ |
| RhCu NW/CF//RhCu NW/CF | 0.56 | 65 | 10.76 | - | *Angew. Chem. Int. Ed.*/2025 | ^[23]^ |
| Pd_nc_-Ni_3_C//Pd_nc_-Ni_3_C | 0.88 | 45 | 7.43 | 24 | *J. Am. Chem. Soc.*/2025 | ^[24]^ |
| Cu NLs@Co(OH)_2_// Cu NLs@Co(OH)_2_ | 0.8 | 122.9 | 13.24 | 120@10 | - | This work |

**Table S4.** **Price^a)^ of input chemicals and products.**

| Products | Price $/ton |
| --- | --- |
| Input chemicals | |
| Formaldehyde | 650 |
| Products | |
| Potassium formate | 1143 |
| Hydrogen | 3900 |
| Oxygen | 242 |
| Ammonia | 477 |

1. The price is taken from online trade market.

Ammonia Prices, Trend, Chart, Demand, Market Analysis, News, Historical and Forecast Data Report 2025 Edition [Ammonia Price Index, Trend, Chart and Forecast 2025](https://www.imarcgroup.com/ammonia-pricing-report)

Bulk Oxygen Prices, Trend, Chart, Demand, Market Analysis, News, Historical and Forecast Data Report 2025 Edition <https://www.imarcgroup.com/bulk-oxygen-pricing-report>

Formaldehyde Prices, Trend, Chart, Demand, Market Analysis, News, Historical and Forecast Data Report 2025 Edition <https://www.imarcgroup.com/formaldehyde-pricing-report>

Chemicals Market Price & Insight, Inorganic Chemicals, Potassium formate Market Price & Analysis 2026 https://www.echemi.com/productsInformation/pid_Seven44555-potassiumformate.html

Hydrogen Prices, Trend, Chart, Demand, Market Analysis, News, Historical and Forecast Data Report 2025 Edition <https://www.imarcgroup.com/hydrogen-pricing-report>

**References**

[1] P. De Luna, C. Hahn, D. Higgins, S. A. Jaffer, T. F. Jaramillo, E. H. Sargent, *Science* **2019**, 364, eaav3506.

[2] Y. Lum, J. E. Huang, Z. Wang, M. Luo, D.-H. Nam, W. R. Leow, B. Chen, J. Wicks, Y. C. Li, Y. Wang, *Nat. Catal.* **2020**, 3, 14.

[3] L. Zhang, Y. Cai, Y. Li, C. Sun, Y. Xiao, Y. Yang, D. Chen, D. Xiao, C.-F. Lee, Y. Wang, *Energy Environ. Sci* **2025**, 18, 2804.

[4] R. Gao, T. Y. Dai, Z. Meng, X. F. Sun, D. X. Liu, M. M. Shi, H. R. Li, X. Kang, B. Bi, Y. T. Zhang, *Adv. Mater.* **2023**, 35, 2303455.

[5] K. Fan, W. Xie, J. Li, Y. Sun, P. Xu, Y. Tang, Z. Li, M. Shao, *Nat. Commun.* **2022**, 13, 7958.

[6] Q. Hu, K. Yang, O. Peng, M. Li, L. Ma, S. Huang, Y. Du, Z.-X. Xu, Q. Wang, Z. Chen, *J. Am. Chem. Soc.* **2023**, 146, 668.

[7] Y. Liu, J. Wei, Z. Yang, L. Zheng, J. Zhao, Z. Song, Y. Zhou, J. Cheng, J. Meng, Z. Geng, *Nat. Commun.* **2024**, 15, 3619.

[8] J. Yu, R. T. Gao, X. Guo, N. Truong Nguyen, L. Wu, L. Wang, *Angew. Chem. Int. Ed.* **2025**, 64, e202415975.

[9] W. Yu, J. Yu, M. Huang, Y. Wang, Y. Wang, J. Li, H. Liu, W. Zhou, *Energy Environ. Sci* **2023**, 16, 2991.

[10] Z. Chang, G. Meng, Y. Chen, C. Chen, S. Han, P. Wu, L. Zhu, H. Tian, F. Kong, M. Wang, *Adv. Mater.* **2023**, 35, 2304508.

[11] J.-Y. Fang, Q.-Z. Zheng, Y.-Y. Lou, K.-M. Zhao, S.-N. Hu, G. Li, O. Akdim, X.-Y. Huang, S.-G. Sun, *Nat. Commun.* **2022**, 13, 7899.

[12] F.-Y. Chen, Z.-Y. Wu, S. Gupta, D. J. Rivera, S. V. Lambeets, S. Pecaut, J. Y. T. Kim, P. Zhu, Y. Z. Finfrock, D. M. Meira, *Nat. Nanotech.* **2022**, 17, 759.

[13] T. Huang, T. Liang, J. You, Q. Huo, S. Qi, J. Zhao, N. Meng, J. Liao, C. Shang, H. Yang, *Energy Environ. Sci* **2024**, 17, 8360.

[14] L. Zhou, X. Chen, S. Zhu, K. You, Z. J. Wang, R. Fan, J. Li, Y. Yuan, X. Wang, J. Wang, *Angew. Chem. Int. Ed.* **2024**, 63, e202401924.

[15] Q. Yang, Y. Bu, S. Pu, L. Chu, W. Huang, X. Zhu, C. Liu, G. Fang, P. Cui, D. Zhou, *Angew. Chem. Int. Ed.* **2024**, 63, e202400428.

[16] J. Lv, A. Cao, Y. Zhong, Q. Lin, X. Li, H. B. Wu, J. Yan, A. Wu, *Nat. Commun.* **2024**, 15, 6675.

[17] J. Li, H. Li, K. Fan, J. Y. Lee, W. Xie, M. Shao, *Chem Catal.* **2023**, 3, 100638.

[18] Q. Yan, R. Zhao, L. Yu, Z. Zhao, L. Liu, J. Xi, *Adv. Mater.* **2024**, 36, 2408680.

[19] G. Zhang, X. Li, K. Chen, Y. Guo, D. Ma, K. Chu, *Angew. Chem. Int. Ed.* **2023**, 62, e202300054.

[20] Q. Gao, H. S. Pillai, Y. Huang, S. Liu, Q. Mu, X. Han, Z. Yan, H. Zhou, Q. He, H. Xin, *Nat. Commun.* **2022**, 13, 2338.

[21] S. An, Z. H. Zhao, J. Bu, J. He, W. Ma, J. Lin, R. Bai, L. Shang, J. Zhang, *Angew. Chem. Int. Ed.* **2024**, 63, e202318989.

[22] R. Javed, B. Zhao, Z. Zafar, Q. Zhao, A. Surulinathan, N. Chen, R. Feng, Y. Zhang, X. Z. Fu, J. L. Luo, *Adv. Mater.* **2025**, 37, e12332.

[23] X. Long, B. Zhao, D. Liu, G. Fu, H. Yang, R. Feng, N. Chen, H. Ding, J. Wu, Y. Liao, *Angew. Chem. Int. Ed.* **2025**, e202503424.

[24] Z. Zafar, B. Zhao, R. Javed, A. Surulinathan, X. Long, M. B. Hussain, N. Chen, R. Feng, Y. Zhang, X.-Z. Fu, *J. Am. Chem. Soc.* **2025**, 147, 40356.
